# Enhanced hybrid microgrid stability with electric vehicle integration using PDA-FOPIID control optimized by tianji’s horse racing algorithm

**DOI:** 10.1038/s41598-025-22993-1

**Published:** 2025-10-30

**Authors:** Hossam Kotb, Ali M. Elkassas, Ashraf Ibrahim Megahed, Muhammad R. Hammad, Kareem M. AboRas

**Affiliations:** Department of Electrical Power and Machines, Faculty of Engineering, Alexandria University, Alexandria, 21544 Egypt

**Keywords:** Cascade controller, (PDA) controller, (FOPIID) controller, EV, LFC, AVR, Tianji’s horse racing optimization, Microgrid, Dual-area interconnected hybrid power system, RES, Energy science and technology, Engineering, Mathematics and computing

## Abstract

Ensuring stable voltage and frequency regulation in hybrid microgrids has become a pressing challenge due to the increasing penetration of renewable energy sources and the integration of electric vehicles. The strong coupling between active and reactive power dynamics often leads to instability, degraded dynamic performance, and difficulties in maintaining tie-line power balance. To address these challenges, this study proposes a novel cascaded controller that combines Proportional-Derivative-Acceleration (PDA) and Fractional-Order Proportional-Integral-Double Integral-Derivative (FOPIID) control strategies, optimized using Tianji’s Horse Racing Optimization (THRO) algorithm. The proposed PDA-FOPIID controller functions as a robust secondary control strategy that effectively decouples voltage and frequency regulation while improving transient response. Optimization through THRO ensures superior performance compared to other well-known metaheuristic algorithms, achieving the lowest Integral Time-Square Error (ITSE) value of 0.0297. Extensive MATLAB/Simulink simulations under diverse operating conditions including step load changes, random fluctuations, multi-step perturbations, and high renewable penetration demonstrate the controller’s effectiveness. Comparative results against several benchmark controllers, namely Proportional-Integral-Derivative (PID), Tilt-Integral-Derivative (TID), Fractional-Order PID (FOPID), PD-(1 + PI), and FOPI-PIDD², confirm that the proposed PDA-FOPIID achieves approximately 48% faster settling time, improved damping, and superior voltage and frequency stability. These findings highlight its potential as a promising solution for enhancing the reliability and resilience of modern hybrid microgrids with high renewable and electric vehicle integration.

**Supplementary Information:**

The online version contains supplementary material available at 10.1038/s41598-025-22993-1.

## Introduction

### Background

 Managing both the terminal voltage of synchronous generators and area frequency remains a significant challenge in electrical power systems. Any deterioration in these parameters can severely impact the lifespan and performance of other system components. To handle minor load disturbances and maintain frequency and voltage within acceptable limits, large-scale power systems employ control devices. Power plants feature two fundamental control loops: the load frequency control loop, which minimizes the gap between actual power generation and demand to regulate frequency, and the automatic voltage regulator loop, which manages reactive power and thereby controls terminal voltage^1^.

For decades, reliable and affordable electricity has been essential to technological progress. However, rising energy demands driven by population growth and development have significantly increased consumption. Historically, the power sector relied on conventional, non-renewable sources. Today, concerns over resource depletion, environmental impact, and the corporation of renewable energy sources (RESs) are driving a shift toward cleaner alternatives^2^. The deployment of energy storage systems has gained notable attention for enhancing renewable-based power networks, supported by research efforts, industry investment, and policy incentives. Similarly, the coordinated integration of electric vehicles (EVs) is being explored for their potential to support grid stability and reliability^3^. To further enhance operational efficiency, advanced optimization techniques such as stochastic methods^4^ and resilient strategies^5^ are being applied. Despite these advancements, RES-based systems face significant challenges, including intermittency, low inertia, and unpredictable loads. While interconnected renewable networks offer advantages, they also introduce greater vulnerability to disturbances and slower dynamic responses^6^. A key issue is the weak inertial response in renewable grids, unlike fossil-fuel-based systems. Wind and solar power, interfaced via power converters, lack the rotating mass needed to deliver adequate inertia^7^, limiting their responsiveness and increasing issues like harmonic distortion^8^. Wind energy integration is growing steadily due to its abundance and the push to lower CO₂ emissions^9^, but its variability can disrupt supply-demand balance, especially during nighttime when Load Frequency Control (LFC) may be insufficient^10,11^.

Electric vehicle (EV) adoption has increased significantly due to efforts to reduce fossil fuel use, emissions, and charging costs^12^. Using vehicle-to-grid (V2G) strategies, EVs can support various grid functions, such as smoothing renewable generation^13^ and providing ancillary services^14,15^. The fast response of EV batteries makes them well suited for frequency regulation, helping to balance power fluctuations and enhance overall system dynamics. Additionally, EV participation in frequency control can provide economic benefits through market engagement. However, integrating EVs into frequency management, particularly in multi-area systems, introduces complexities that challenge traditional load balancing methods and require more adaptive control approaches. Previous studies have also investigated advanced energy storage technologies, including ultra-batteries^16^ and redox flow batteries, in both deregulated^17^ and conventional^18^ frequency control systems. The combined performance of EVs with other renewable sources has been analyzed, showing their effectiveness^19,20^. Despite this progress, there remains a gap in research addressing a combined voltage regulation and load frequency control framework for EVs, indicating the need for further investigation.

In addition to the integration of electric vehicles and photovoltaic systems, several studies have highlighted the impact of ambient factors on power system performance. Variations in temperature, humidity, wind, and seasonal or spatial conditions can significantly influence transmission limits, generation efficiency, and overall system stability. For instance, decentralized methods for calculating power transfer limits that account for spatial and seasonal differences in ambient conditions have been proposed, emphasizing the need to consider environmental factors alongside renewable integration for more accurate and reliable system operation^21^.

### Literature review

A wide range of controllers has been proposed for load frequency control^22–24^, including traditional, predictive, fuzzy logic, neural network, fractional-order, and hybrid approaches. Various combinations of proportional, integral, derivative, and filtered derivative elements have also been explored. In^25^, a proportional–integral controller designed for electric vehicles was introduced, although it faced stability issues under communication delays. To improve performance in systems with renewable energy integration, the Tilt-Integral-Derivative Filter controller was optimized using the Differential Evolution algorithm^25^. Reference^26^ combined proportional–integral, tilt–derivative, and filter controllers, while^27^ proposed a hybrid genetic algorithm–particle swarm optimization approach to stabilize frequency in power networks.

For multi-generation systems, a fractional-order regulator tuned by the Imperialist Competitive Algorithm was presented in^28^, effectively managing multiple step changes in load and generation. In dual-area systems, cascading fractional-order proportional–integral–derivative and fuzzy logic controllers have been used for frequency regulation^29^, while^30^ employed the Grey Wolf Optimizer for load frequency control design. A fractional-order PID controller with a fractional-order filter was tuned using the Sine–Cosine Algorithm in^31^. Reference^32^ introduced an optimal self-tuning fractional-order fuzzy regulator for dual-area hybrid systems, with parameters optimized via the Pathfinder Algorithm. In^33^, a single-region pumped storage system compliant with IEEE standards was developed using a fractional-order PID-based load frequency controller, optimized with the Chaos Particle Swarm Optimization method. Reference^34^ compared different controllers for shipboard microgrids with multiple energy sources, considering communication delays and optimized using the Jellyfish Search Optimizer. In^35^, proportional–integral controller parameters were tuned using Harris Hawk Optimization, while^36^ proposed a fractional-order integral–tilt–derivative–nonlinear controller for multisource power grids with capacitive storage, optimized via a hybrid algorithm. Controlled electric vehicles with Tilt-Integral-Derivative Filter regulators were optimized using the Bee Colony Optimizer in^37^, and virtual inertia concepts were enhanced using particle swarm optimization in^38^. To address automatic generation control challenges in interconnected grids^39^, introduced an ultra-capacitor-based energy storage solution. An enhanced fractional-order Tilt-Integral-Derivative controller was developed using the Pathfinder Optimizer in^40^. Amil et al.^41^ proposed multi-fractional-order PID regulators optimized using the Jellyfish Search Optimizer for hybrid grid frequency stability. Reference^42^ employed the Imperialist Competitive Algorithm to tune regulator gains, while Mohamed et al.^43^ introduced a tilt-filtered derivative fractional-order controller optimized via the Artificial Hummingbird Algorithm. The Slap Swarm Algorithm was applied to tune PID gains for two-area systems in^44^, and the Butterfly Optimization method was used in^45^ to design a dual-stage controller. Reference^46^ recommended cascaded fractional-order integral–derivative controllers with filters for automatic generation control in hybrid grids. Collectively, these studies highlight the strong influence of controller type and optimization method on transient grid performance.

Reference^47^ proposed a fuzzy logic interface using a compensation method to maintain functionality in the presence of actuator faults and nonlinearities within Markov jump networks. A neural network-based fault-tolerant framework for Markovian jump systems was presented in^48^. The Gorilla Troops Optimizer was applied in^49^ to regulate power flow with integrated Thyristor-Controlled Series Compensation units, improving voltage stability, reducing fuel costs, and minimizing emissions. In^50^, the Coyote Optimization Algorithm was used to fine-tune proportional–integral and proportional–derivative controller combinations for multi-area frequency regulation. A type-2 fuzzy PID regulator, optimized using the Water Cycle Algorithm, was developed in^51^ for multi-region grids with generation rate constraints. Reference^52^ applied the Arithmetic Optimization Algorithm to tune a fuzzy PID controller while considering AC transmission limitations and the effects of high-voltage DC links. In^53^, a hybrid controller combining neural networks with rapid traversal filters was proposed to analyze voltage and frequency interactions in a single-area system, while^54^ used particle swarm optimization to tune PID parameters for an integrated voltage–frequency control model. Further studies^55–58^ continued to explore such interactions. Although PID-based controllers are widely used, their robustness often decreases under dynamic uncertainties in power generation. Incorporating advanced control strategies can significantly enhance stability under parameter variations. For example^59^, demonstrated that a hybrid fuzzy proportional–derivative controller with a cascaded PI–PD setup outperformed both fuzzy PID and conventional PID controllers in multi-microgrid frequency control applications, and^60^ showed that a cascaded PI–Tilt–Integral–Derivative controller provided superior performance compared to standalone TID and classical PID controllers in interconnected power systems.

In recent years, artificial intelligence (AI)-based control strategies have attracted increasing attention in the field of microgrid operation and stability enhancement. Data-driven and learning-based approaches are being explored to address the challenges of nonlinearities, uncertainties, and communication delays. For instance, a recent study proposed a data-driven method for online gain scheduling of distributed secondary controllers in time-delayed microgrids, employing deep reinforcement learning (DRL) to dynamically adapt controller parameters and improve resilience. Such AI-based methods demonstrate strong adaptability and decision-making capabilities; however, they often involve high computational requirements and complex training processes. In contrast, the present study focuses on a cascaded PDA–FOPIID controller optimized via Tianji’s Horse Racing Optimization, which offers a balance between computational efficiency and robust dynamic performance^61^.

Furthermore, recent studies have focused on developing advanced controllers to enhance AGC performance in modern interconnected power systems with renewable penetration and virtual inertia support. For instance, Vidyarthi et al.^62^ proposed a chaos quasi-opposition-based sea-horse optimized cascaded tilted controller combined with deep learning techniques to improve frequency regulation under cyber-attack scenarios. Similarly, a modified MPC-based approach was introduced in^63^, which incorporated virtual inertia to mitigate the adverse impacts of renewable intermittency in multi-area systems. Further extensions include cascaded Tilt-MPC controllers^64^ and hybrid MPC + PIDN structures^65^, both optimized using advanced meta-heuristics and tested under high renewable integration. While these studies demonstrate significant improvements in frequency control, they primarily concentrate on AGC dynamics alone and do not explicitly address the coupled interaction between LFC and AVR in hybrid systems. To overcome this gap, the present work introduces a novel PDA–FOPIID controller optimized by THRO, which simultaneously handles LFC–AVR coupling and achieves superior dynamic performance compared with existing strategies.

In reviewing the existing literature, it was observed that not all studies provide a detailed consideration of electric vehicle integration, and many rely on conventional rather than modern optimization algorithms. Moreover, nonlinearities such as the generation rate constraint (GRC) and governor dead band (GDB) have not been comprehensively addressed, and only a limited number of works have jointly analyzed the integration of load frequency control (LFC) with automatic voltage regulation (AVR). These limitations highlight clear research gaps that motivated the present study.

This inspired the authors to propose a novel integrated PDA–FOPIID controller for the combined AVR–LFC model within a two-area hybrid system, aiming to improve both frequency and voltage stability. Unlike most existing studies that rely on traditional or fractional-order controllers, the proposed approach leverages the PDA–FOPIID structure, optimized using the Tianji’s Horse Racing Optimization (THRO) algorithm^66^, to enhance robustness and efficiency under the increased complexity and severe disturbances of modern interconnected systems.

### Contribution

This study introduces a novel PDA-FOPIID controller aimed at improving the stability of frequency and voltage in interconnected dual-area hybrid power systems, particularly under power disturbances and varying operating conditions. To achieve optimal tuning, the Tianji’s Horse Racing Optimization (THRO) algorithm is employed, offering an efficient and adaptive approach to complex nonlinear control problems. The main contributions of this work can be summarized as follows:

- Design of a PDA-FOPIID regulator to enhance frequency and voltage regulation in interconnected hybrid systems.
- Adoption of the THRO algorithm for precise and efficient controller parameter tuning.
- Comparative analysis demonstrating the superior performance of the proposed method over advanced optimizers such as GTO, PSO, WHO, and GBO.
- Benchmarking against conventional and advanced controllers, including PID^67^, TID^68^, FOPID^69^, PD-(1 + PI)^70^, and FOPI-PIDD2^71^.
- Extensive evaluation under diverse scenarios, including step load changes, multi-step disturbances, random fluctuations, and high renewable energy penetration.

The structure of the study is as follows:

- **Phase 2** provides a detailed description of the system architecture along with an overview of its key components.
- **Phase 3** describes the suggested THRO algorithm and outlines the configuration of the designed PDA-FOPIID controller.
- **Phase 4** presents the results of simulation analysis under a variety of test cases.
- **Phase 5** summarize the research by highlighting key findings and recommendations for future work.

## Hybrid system modeling with electric vehicle participation

**Fig. 1:**
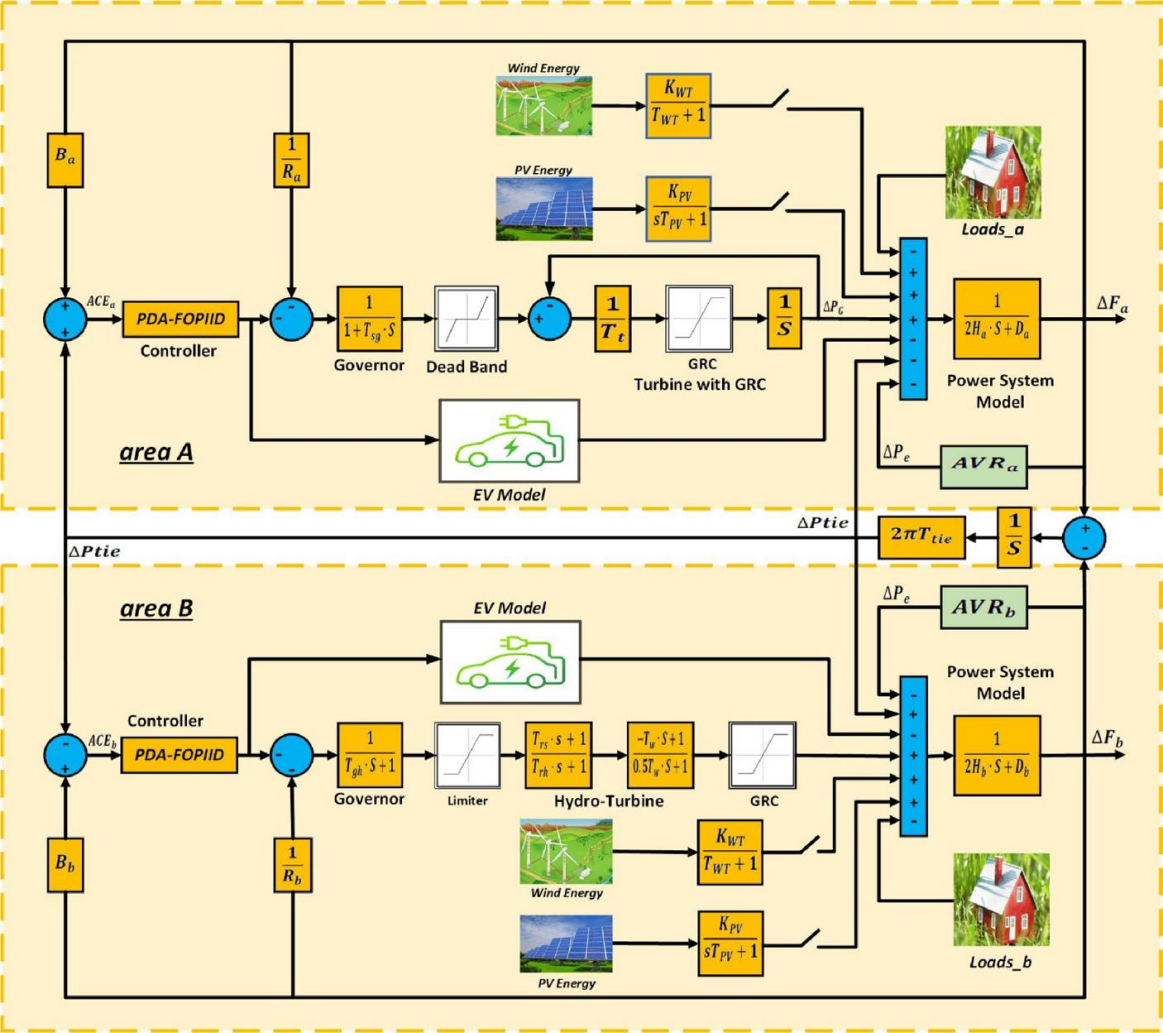
Dual area hybrid power system.

To examine the effectiveness of the presented THRO-based PDA-FOPIID regulator, a two-area interconnected power system, adapted from^43^, is utilized as a case study. Models of selected power grid and the proposed PDA-FOPIID controller, including the integration of electric vehicles (EVs), is illustrated in Fig. 1. The network includes two regions area A and B. Area A includes local loads and thermal power generation, while area B comprises local loads and hydroelectric generation. Depending on the specific evaluation scenario, photovoltaic (PV) and wind renewable energy sources are implemented in either or both areas to simulate realistic operating conditions. Moreover, Automatic voltage regulator (AVR) systems are connected in both areas to maintain terminal voltage stability. EVs are assumed to be uniformly distributed across the entire network, supporting both frequency and voltage regulation.

### Thermal energy source modeling

The thermal power plant situated in area A includes a governor with a dead band (GDB) with a 0.5% backlash, and a Steam turbine featuring a 10% per unit per minute generation rate constraint (GRC) (equivalent to 0.0017 pu.MW/s). The corresponding transfer function models for these components are expressed as follows^72^:

1 $$\:Governor=\frac{1}{1+{T}_{sg}S}$$

2 $$\:thermal\:turbine=\frac{1}{1+{T}_{t} \cdot S}$$

Here, $\:{T}_{sg}$ denotes the steam governor’s time constant, set at 0.08 s. Additionally, $\:{T}_{t}$​ refers to the thermal turbine’s time constant, which is set to $\:0.3\:seconds$.

### Hydropower generation system model

The hydraulic power plant in area B includes a transient droop compensator, a governor, and a hydropower turbine with 270% per unit per minute generation rate constraint (GRC) (equivalent to 0.045 pu.MW/s). The corresponding transfer function models for these components are given as follows^72^:

3 $$\:governor=\frac{1}{1+{T}_{gh} \cdot S}$$

4 $$\:Transient\:droop\:compensator=\frac{{T}_{rs} \cdot \:s\:+\:1}{{T}_{rh} \cdot \:s\:+\:1}$$

5 $$\:Hydraulic\:turbine=\frac{{-T}_{w} \cdot S+1}{{0.5T}_{w} \cdot S+1}$$

Here, $\:{T}_{gh}$​ denotes the hydraulic governor’s time constant, set at 41.6 s; $\:{T}_{rs}$ refers to the hydro turbine speed governor’s reset interval, with a value of $\:5\:seconds$; $\:{T}_{rh}$​ represents the time constant of the transient droop compensator, assigned a value of $\:0.513\:seconds$; and $\:{T}_{w}$​ is the standard water start-up time for the hydro turbine, valued at $\:1\:seconds$.

The frequency bias coefficients, $\:{\beta\:}_{a}$ and $\:{\beta\:}_{b}$​, are assigned values of 0.4249. The speed regulation parameters of the governors for the thermal and hydro units, represented by $\:{R}_{a}$ and $\:{R}_{b}$​, are both set to 2.4. The inertia constants of power systems A and B, denoted as $\:{H}_{a}$ and $\:{H}_{b}$ ​, are 0.0833 p.u.s each, while the damping coefficients, $\:{D}_{a}$ and $\:{D}_{b}$​, are both set to 0.00833 p.u./Hz. Appendix 1 provides the corresponding parameter values for power systems A and B, as well as the tie-line.

### Photovoltaic (PV) farm generation model

The PV system’s generated power $\:{P}_{out\_PV}$ is primarily influenced by two key factors: environmental temperature $\:{T}_{O}$ and solar irradiation $\:{\theta\:}_{S}$ over the surface of the PV array. The result of $\:{P}_{out\_PV}$ can be expressed as follows^73^:

6 $$\:{P}_{out\_PV}={P}_{STC} \cdot \left(\frac{{\theta\:}_{S}}{{\theta\:}_{STC}}\right) \cdot 1+(\alpha\:{(T}_{O}-{T}_{R})) \cdot \eta\:\text{{M}}$$

Here, $\:{P}_{STC}$​ refers to the typical output power measured at Standard Test Conditions (STC), while $\:{\theta\:}_{S}$​ represents the solar irradiance at STC, typically valued at 1000 W/m². The parameter α\alphaα is the temperature coefficient, and $\:{T}_{R}$​ is the reference temperature, generally taken as 25 °C. Additionally, $\:\eta\:\text{{\rm\:M}}$ ​ denotes the maximum power point tracking (MPPT) efficiency. Since PV output power is linearly dependent on solar irradiation, this research adopts a basic first-order dynamic model to represent the PV system, as described in Eq. (8). The model includes a unity gain, represented by $\:{K}_{PV}$​, and a $\:1.3\:seconds$ time constant, represented as $\:{T}_{PV}$​^73^.

7 $$\:PV\:model=\frac{{K}_{PV}}{1+{T}_{PV} \cdot S}$$

Furthermore, the solar irradiance input used in the PV system model is based on real-world data collected in July 2020 from a functioning PV station featuring a 1.5 GW capacity, situated in Egypt, Aswan, horizontal coordinate: 24.08° N, longitude: 32.89° east.

### Wind farm power generation model

The power output of a wind turbine generator $\:{P}_{out\_W}$ can be calculated using the standard wind power equation^73^:

8 $$\:{P}_{out\_W}=0.5 \cdot {C}_{P} \cdot \rho\: \cdot {A}_{t} \cdot {V}_{W}^{3}$$

Here, $\:{P}_{out\_W}$ denotes the wind turbine’s generated power, $\:{C}_{P}$​ is the power coefficient typically adjusted to extract maximum power from the wind $\:\rho\:$ represents density of air in kg/m³, $\:{A}_{t}$​ is the swept area of the rotor in m², and $\:{V}_{W}^{}$​ is the nominal speed of the wind in m/s. For this study, the wind power generation system is modeled using a basic first-order dynamic model, as given in Eq. (10). The model of the wind turbine, in its linear form, employs a unit gain, represented by $\:{K}_{W}$​, and a 1.5 s time constant of, represented by $\:{T}_{W}$​^73^.

9 $$\:Wind\:model=\frac{{K}_{W}}{1+{T}_{W} \cdot S}$$

Actual wind speed data is also employed in this analysis gathered in April 2020 from a representative wind farm in the vicinity of Zafarana, Egypt a latitude: 29.23° N, longitude: 32.59° east, the site experiences wind speeds ranging from 6 up to 14 m/s.

### Electric vehicle (EV) system model

**Fig. 2:**
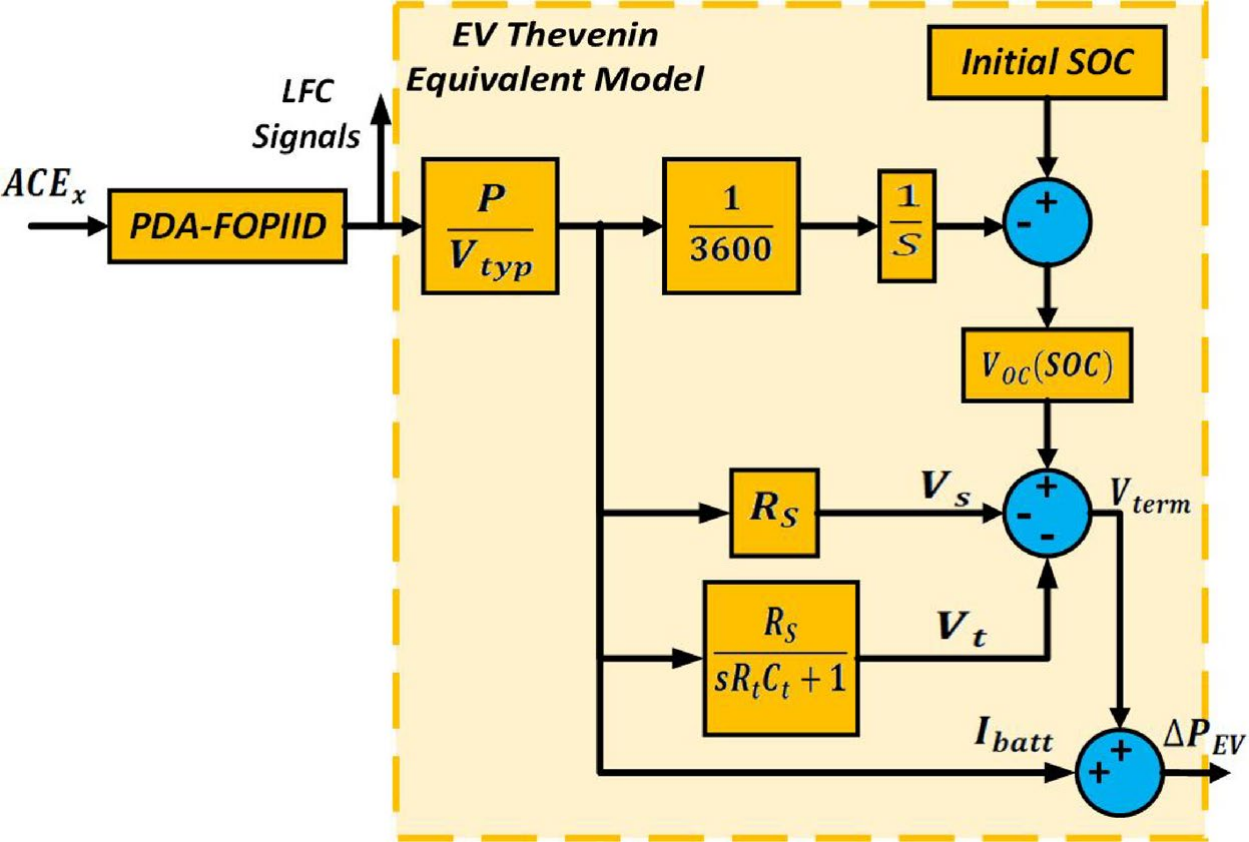
Thevenin equivalent model of the electric vehicle (EV).

Recent advancements have enabled electric vehicles (EVs) to actively participate in frequency regulation. Utilizing EV batteries for this purpose offers improved charge/discharge control while reducing the need for additional energy storage infrastructure. To implement such frequency support capabilities, it is essential to model the internal battery characteristics within the load frequency control (LFC) framework. This study assumes that EVs are uniformly distributed across the networked power networks. As illustrated in Fig. 2 and detailed in^43^, the commonly adopted equivalent circuit according to Thevenin’s theorem is used to model EV battery behavior. In this model, there is an open-circuit voltage source $\:{V}_{OC\left(SOC\right)}$, which varies with the battery’s state of charge $\:\left(SOC\right)$. The circuit also comprises a parallel RC network $\:{R}_{T},\:{C}_{T}$ and a series resistance $\:{R}_{S}$, with values set to $\:0.047\:\varOmega\:,\:703.6\:F$, and $\:0.074\:\varOmega\:$, respectively. The $\:RC$ branch simulates transient overvoltage effects. The final voltage $\:{V}_{term}$​ at the EV output is determined by deducting the voltage drops $\:{V}_{s}$​ and $\:{V}_{t}$ from the open-circuit voltage $\:{V}_{OC\left(SOC\right)}$, as depicted in Fig. 2. The relationship between the SOC and $\:{V}_{OC}$​ is further described by the next equation, which reflects the electrochemical behavior of the installed EV batteries^43^.

10 $$\:{V}_{OC\left(SOC\right)}={V}_{typ}+S\frac{RT}{F}\text{ln}\left(\frac{SOC}{{C}_{Typ}-SOC}\right)$$

In this context, $\:{V}_{typ}$ represents the nominal voltage of the EV battery, valued at $\:364.8\:V$, while $\:{C}_{Typ}$​ denotes its typical capacity, set at 66.2 Ah. The parameter SSS defines the sensitivity of the open-circuit voltage $\:{V}_{OC\left(SOC\right)}$ to the state of charge. $\:F\:$is constant of Faraday, temperature is represented by $\:T$, and $\:R$ is the ideal gas constant. The combined constant term $\:\frac{RT}{F}$​ has a value of $\:0.02612$. The minimum and maximum allowable state-of-charge $\:\left(SOC\right)$ levels for the batteries of EV are set at 10% and 95%, respectively. The energy capacity of each EV battery is assumed to be 24.15 kWh.

### Automatic voltage regulator (AVR)

**Fig. 3:**
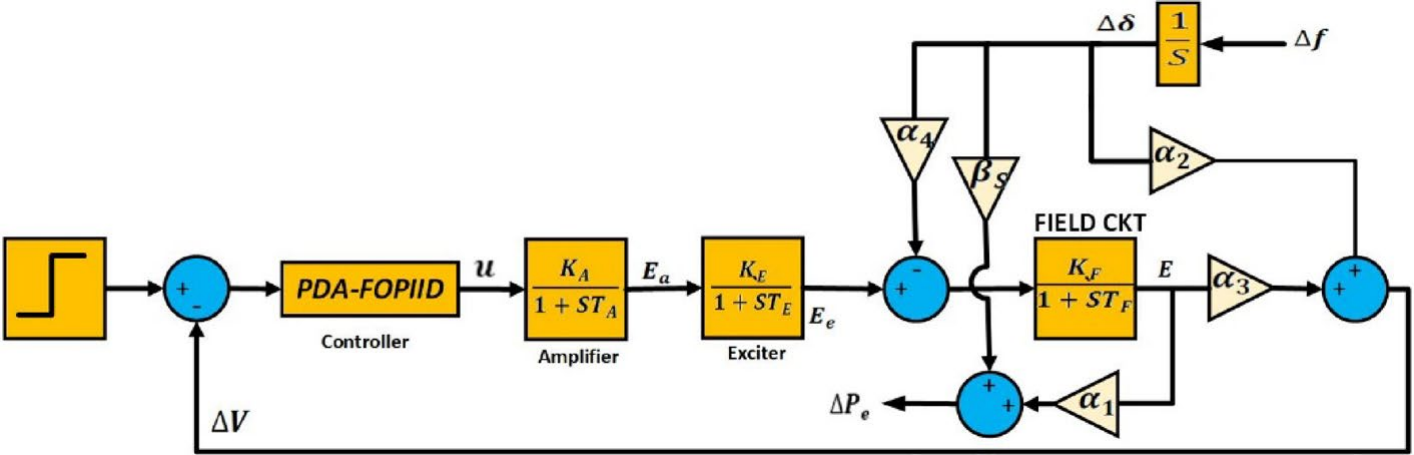
AVR model.

AVR system objects to minimize the losses of reactive power resulting from discrepancy between the target reference voltage and the exciter terminal voltage $\:{E}_{e}$​. Variations in the generator’s reactive power load cause changes in its terminal voltage $\:{E}_{t}$​. As shown in Fig. 2, this terminal voltage is measured using a single-phase instrument transformer, producing a known voltage signal $\:\varDelta\:V$ or $\:{E}_{S}$​, which is compared to a predefined reference voltage $\:{V}_{ref}$​. The resulting error signal is amplified to produce a control voltage $\:{E}_{a}$​​, which regulates the exciter field and, in turn, adjusts the exciter’s terminal voltage. This process directly influences the excitation current of the generator and alters the induced electromotive force $\:\left(emf\right)$[73]. The AVR system’s schematic is illustrated in Fig. 3, while a detailed mathematical representation is provided in Appendix 2.

The AVR system is composed of four primary components: the amplifier, exciter, generator field, and sensor. The terminal voltage is constantly monitored and compared against a preset reference voltage. The generated error signal is then input to the PDA-FOPIID controller, which produces the control signal. The signal is then boosted and sent to the excitation system to modulate the generator’s field current. Notably, even minor frequency deviations can lead to variations in (δ) the angle of the rotor, influencing the delivered active power, which can be formulated as below^74,75^:

11 $$\:\varDelta\:{P}_{e}={P}_{S}\varDelta\:\delta\:+{K}_{1}E$$

The change in terminal voltage is given as,

12 $$\:\varDelta\:V={K}_{2}\varDelta\:\delta\:+{K}_{3}E$$

The transfer function of generator field is given as,

13 $$\:E=\:\frac{{K}_{F}}{1+{sT}_{F}}\left({E}_{e}-{K}_{4}\varDelta\:\delta\:\right)$$

The dynamic interaction between the automatic voltage regulator (AVR) and load frequency control (LFC) systems is relatively weak, allowing voltage and frequency regulation to be managed independently within each area. However, despite their separate operation, the AVR’s control over terminal voltage has a notable influence on the system’s real power output^68^, thereby exerting a direct and significant impact on the LFC loop. Figure 3 depicts the AVR control loop and identifies the coupling coefficients that characterize the interplay among critical system variables. These coefficients represent: how slight variations in the stator electromotive force (emf) affect real power output (α_1_); how small rotor angle deviations influence terminal voltage (α_2_); how changes in rotor angle affect stator emf (α_4_); and how fluctuations in stator emf impact rotor angle (α_3_). The specific values of these coefficients are provided in Appendix 3.

## Problem formulation and control design approach

The design methodology of the proposed PDA-FOPIID controller is detailed in this section, with its coefficients tuned by the THRO algorithm to capably regulate the issues encountered in load frequency control (LFC) and automatic voltage regulator (AVR). Previous studies have shown that traditional controllers often struggle under system uncertainties. By integrating the strengths of the PDA-FOPIID structure, the proposed approach enhances both control performance and robustness in frequency and voltage regulation. To clearly articulate this design process, the section is divided into two subsections: the first introduces the THRO algorithm used for parameter optimization, while the second details the specific architecture and implementation of the PDA-FOPIID controller.

### Tianji’s horse racing optimizer (THRO)

#### Inspiration

**Fig. 4:**
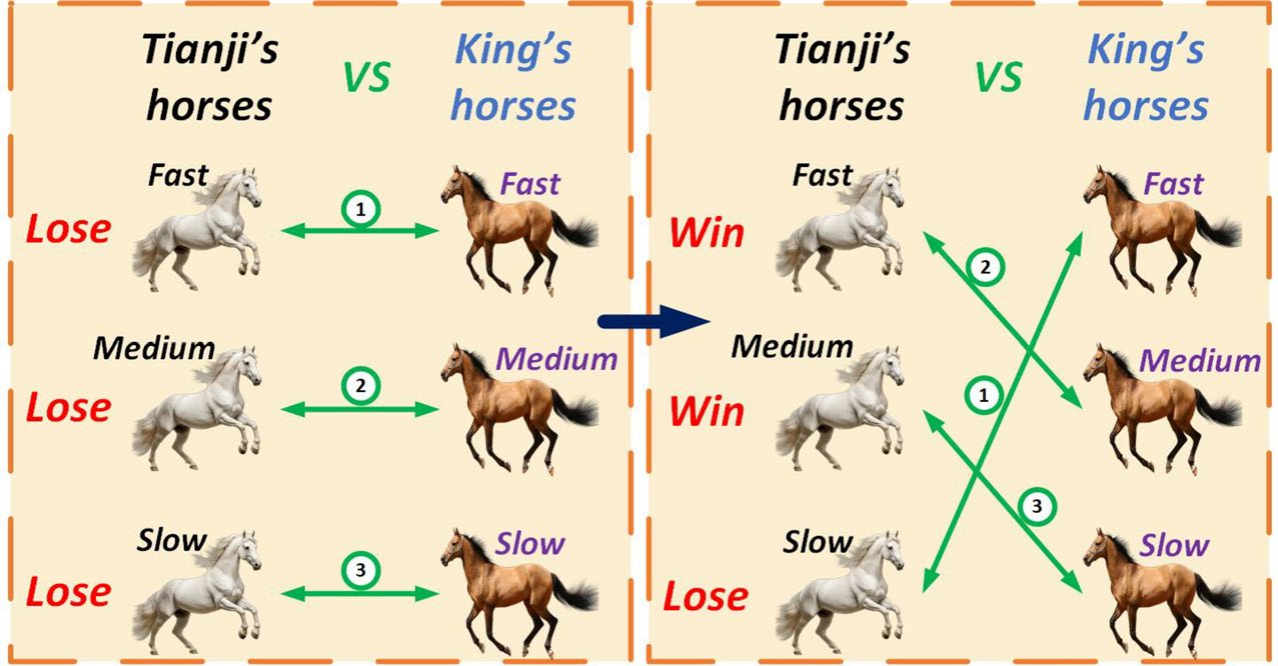
The strategic horse race plan of Tianji.

The story of Tianji’s horse racing, from China’s Spring–Autumn Period, illustrates how strategy can turn disadvantages into strengths^76,77^. This tale inspired the THRO-based global optimization approach, with its key events linked to the algorithm’s mechanisms (Fig. 4). Tianji, a general of the Qi State, often lost races against the king, whose horses were superior in every class. Guided by his advisor Sunbin, Tianji changed strategy: he sacrificed the first round by matching his slowest horse against the king’s fastest, then secured two wins by pairing his fastest with the king’s medium horse and his medium with the king’s slowest. Winning two of three races, Tianji showed the power of adaptive thinking. In the THRO algorithm, this translates to exploration (slow horse), exploitation (fast horse), and balance (medium horse), with fitness gaps guiding the choice of strategy. These adaptive dynamic forms the foundation of its global optimization framework.

#### Competition

In the THRO approach, consider two separate groups: one representing horses of Tianji and the other representing the king’s horses, with each population containing $\:n$ horses. Tianji’s horses are represented as:

14 $$\:{X}_{T}=\left[\begin{array}{c}{x}_{T1}\\\:\begin{array}{c} \vdots \\\:{x}_{Ti}\end{array}\\\:\begin{array}{c} \vdots \\\:{x}_{Tn}\end{array}\end{array}\right]=\left[\begin{array}{ccccc}{x}_{T1}^{1}&\:\cdots\:&\:{x}_{T1}^{j}&\:\cdots\:&\:{x}_{T1}^{d}\\\: \vdots &\:\ddots\:&\: \vdots &\:\ddots\:&\: \vdots \\\:{x}_{Ti}^{1}&\:\cdots\:&\:{x}_{Ti}^{j}&\:\cdots\:&\:{x}_{Ti}^{d}\\\: \vdots &\:\ddots\:&\: \vdots &\:\ddots\:&\: \vdots \\\:{x}_{Tn}^{1}&\:\cdots\:&\:{x}_{Tn}^{j}&\:\cdots\:&\:{x}_{Tn}^{d}\end{array}\right]$$

Where ​$\:{x}_{Ti}$ denotes the $\:i-th$ horse in Tianji’s population, each horse possesses various characteristics, including breed, physical condition, and age, that impact its running speed. The term $\:{{x}_{}}_{Ti}^{j}$​ represents the $\:j-th$ attribute of the $\:i-th$ horse, with $\:d$ being the total number of attributes. In the context of a minimization objective, the objective function reflects the speed of the horse, where a lower value signifies a faster horse.

In the same manner, the horses of the king can be represented as:

15 $$\:{X}_{K}=\left[\begin{array}{c}{x}_{K1}\\\:\begin{array}{c} \vdots \\\:{x}_{Ki}\end{array}\\\:\begin{array}{c} \vdots \\\:{x}_{Kn}\end{array}\end{array}\right]=\left[\begin{array}{ccccc}{x}_{K1}^{1}&\:\cdots\:&\:{x}_{K1}^{j}&\:\cdots\:&\:{x}_{K1}^{d}\\\: \vdots &\:\ddots\:&\: \vdots &\:\ddots\:&\: \vdots \\\:{x}_{Ki}^{1}&\:\cdots\:&\:{x}_{Ki}^{j}&\:\cdots\:&\:{x}_{Ki}^{d}\\\: \vdots &\:\ddots\:&\: \vdots &\:\ddots\:&\: \vdots \\\:{x}_{Kn}^{1}&\:\cdots\:&\:{x}_{Kn}^{j}&\:\cdots\:&\:{x}_{Kn}^{d}\end{array}\right]$$

Let $\:{x}_{Ki}$ represent the $\:i-th$ horse in the population of the king.

In the original tale, each side used only three horses, but the THRO algorithm generalizes this idea to n horses per side (*n* > 3). The horses are ranked from fastest to slowest, corresponding to ascending fitness values in a minimization problem, and divided into $\:n$ classes. Each iteration consists of n rounds, where one horse from Tianji’s team races against the corresponding horse from the king’s team, after which both are removed. For this extended case, the THRO algorithm applies five strategic competition methods as described in^78^.

##### Scenario 1

When Tianji’s slowest horse outperforms the king’s slowest, it is chosen to race and secures a win. To sustain this advantage, the algorithm updates Tianji’s slowest horse using the traits of his fastest one, while also considering the overall quality gap between the two populations. The updated formulation for Tianji’s slowest horse is given as follows.

16 $$\:\left\{\begin{array}{c}{v}_{Tsi}(t+1)=\left(p\times\:{x}_{Tsi}\left(t\right)+\left(1-p\right)\times\:{x}_{Tf}\left(t\right)+R\times\:\left({x}_{Tf}\left(t\right)-{x}_{Tsi}\left(t\right)+p\times\:\left(\overline{{x}_{T}}\left(t\right)-\overline{{x}_{K}}\left(t\right)\right)\right)\right)\times\:\alpha\:+\beta\:\\\:Tsi=Tsi-1\end{array}\right.$$

17 $$\:\propto\:=1+round(0.5\times\:\left(0.5+\text{r}\text{a}\text{n}\text{d}\right))\times\:{n}_{1}$$

18 $$\:{\upbeta\:}=round(0.5\times\:\left(0.1+\text{r}\text{a}\text{n}\text{d}\right))\times\:{n}_{2}$$

19 $$\:p=1-\frac{t}{T}$$

20 $$\:R=L\times\:B$$

21 $$\:L=\frac{u \cdot \sigma\:}{{\left|v\right|}^{\frac{1}{b}}}$$

22 $$\:\sigma\:={\left(\frac{{\Gamma\:}(1+\text{b})\times\:\text{sin}\left(\frac{\pi\:b}{2}\right)}{{\Gamma\:}\left(\frac{1+\text{b}}{2}\right)\times\:b\times\:{2}^{\frac{b-1}{2}}}\right)}^{\frac{1}{b}}$$

23 $$\:B=[{b}_{1},\dots\:,\:{b}_{K},\dots\:,\:{b}_{d}]$$

24 $$\:b\left(k\right)=\left\{\begin{array}{c}1\:\:\:\:\:if\:k==g\left(l\right)\\\:0\:\:\:\:\:\:\:\:\:\:\:\:\:\:\:\:\:\:\:\:\:else\:\end{array}\right.$$

25 $$\:g=randperm\left(d\right)$$

26 $$\:l=1,\dots\:,\:\left[\text{s}\text{i}\text{n}\left(\frac{\pi\:{r}_{1}}{2}\right)\times\:d\right]$$

Let $\:{x}_{Tsi}$​ ​ denote Tianji’s slowest horse, where $\:Tsi$ is its index. Variables $\:{n}_{1},\:{n}_{2},\:u\:and\:v$ follow a standard normal distribution. $\:{x}_{Tf}$ represents Tianji’s fastest horse, while $\:{x}_{T}$​ and $\:{x}_{K}$​ are the mean standards of Tianji’s and the king’s populations, respectively. The coefficient $\:p$ is a weighting factor, $\:\varGamma\:$ is the standard gamma function with $\:b=1.5$, and the running factor $\:R$ applies Lévy flights across random dimensions, enabling both short adjustments and long jumps for global exploration and local escape.

In Eq. (16), the term $\:p\times\:{x}_{Tsi}\left(t\right)+R\times\:\left({x}_{Tf}\left(t\right)-{x}_{Tsi}\left(t\right)\right)\:$moves the slowest horse closer to the fastest, while ($\:\left(1-p\right)\times\:{x}_{Tf}\left(t\right)$ amplifies the fastest horse’s influence. The term $\:p\times\:\left({x}_{T}\left(t\right)-{x}_{k}\left(t\right)\right)$ reflects the quality gap between the two populations, guiding the update direction. A mutation parameter β\betaβ preserves diversity and search efficiency. Finally, the king’s slowest horse is updated in response to Tianji’s, using the following rule:

27 $$\:\left\{\begin{array}{c}{v}_{Ksi}(t+1)=\left(p\times\:{x}_{Ksi}\left(t\right)+\left(1-p\right)\times\:{x}_{Tsi}\left(t\right)+R\times\:\left({x}_{Tsi}\left(t\right)-{x}_{Ksi}\left(t\right)+p\times\:\left(\overline{{x}_{T}}\left(t\right)-\overline{{x}_{K}}\left(t\right)\right)\right)\right)\times\:\alpha\:+\beta\:\\\:Ksi=Ksi-1\end{array}\right.$$

**Fig. 5:**
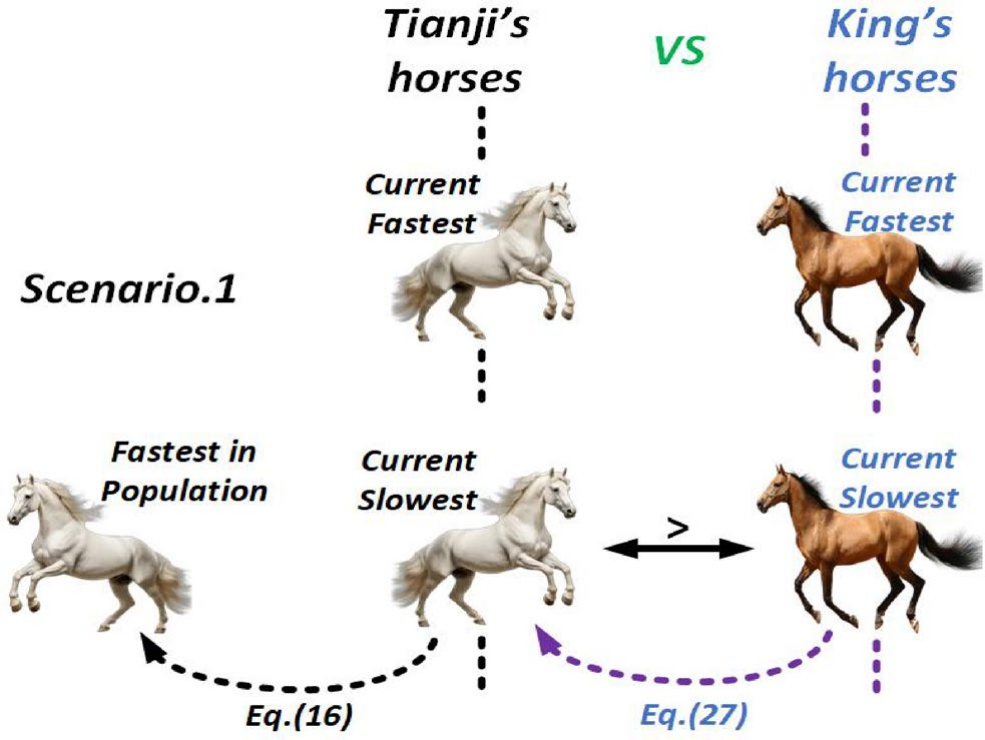
THRO Competition Approach in Scenario 1.

In Eq. (27), $\:{x}_{Ksi}$​ ​ is updated toward $\:{x}_{Tsi}$, considering both the overall quality gap and $\:{x}_{Tsi}$​’s performance, thereby improving the balance between local and global search. Figure 5 illustrates the THRO strategy for Scenario 1, where “>” indicates the faster horse, black double arrows show competing horses, and dashed arrows denote updates based on the pointed horse.

##### Scenario 2

When Tianji’s slowest horse cannot outperform the king’s, it is deliberately matched against the king’s fastest horse. Though this round is lost, the strategy sacrifices the weakest to neutralize the strongest. In this case, since Tianji’s slowest horse is inferior to all of the king’s, it is replaced by randomly selecting another from Tianji’s population. The replacement rule is given as follows.

28 $$\:\left\{\begin{array}{c}{v}_{Tsi}(t+1)=\left(p\times\:{x}_{Tsi}\left(t\right)+\left(1-p\right)\times\:{x}_{Tr1}\left(t\right)+R\times\:\left({x}_{Tr1}\left(t\right)-{x}_{Tsi}\left(t\right)+p\times\:\left(\overline{{x}_{T}}\left(t\right)-\overline{{x}_{K}}\left(t\right)\right)\right)\right)\times\:\alpha\:+\beta\:\\\:Tsi=Tsi-1\end{array}\right.$$

Let $\:{x}_{Tr1}$​ be a horse randomly chosen from Tianji’s population. In Eq. (28), $\:{x}_{Tsi}$ is updated toward $\:{x}_{Tr1}$​, while considering the strength disparity between the two populations and the quality of $\:{x}_{Tr1}$​. Meanwhile, to preserve his advantage, the king replaces his current fastest horse using the best performer in his population. The replacement rule is:

29 $$\:\left\{\begin{array}{c}{v}_{Kfi}(t+1)=\left(p\times\:{x}_{Kfi}\left(t\right)+\left(1-p\right)\times\:{x}_{Kf}\left(t\right)+R\times\:\left({x}_{Kf}\left(t\right)-{x}_{Kfi}\left(t\right)+p\times\:\left(\overline{{x}_{T}}\left(t\right)-\overline{{x}_{K}}\left(t\right)\right)\right)\right)\times\:\alpha\:+\beta\:\\\:Kfi=Kfi+1\end{array}\right.$$

**Fig. 6:**
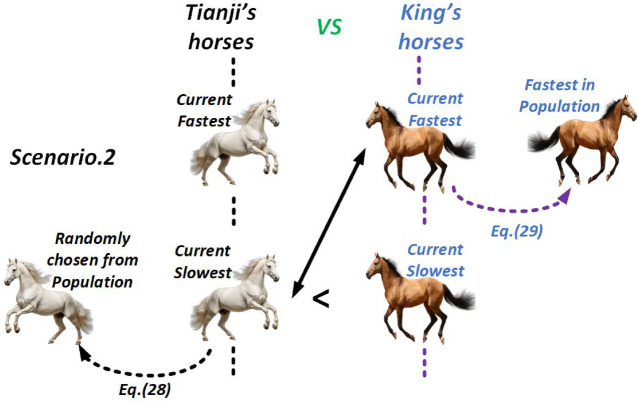
THRO Competition Approach in Scenario 2.

Here, $\:{x}_{Kf}$​ denotes the fastest horse in the king’s team, and $\:Kfi$ represents the index of the fastest currently horse of the king. Figure 6 illustrates the THRO algorithm’s challenging strategy for scenario 2.

##### Scenario 3

If Tianji’s slowest horse matches the king’s, but his fastest outperforms the king’s, Tianji uses his fastest horse to win the round. To maintain this lead, the algorithm updates Tianji’s fastest horse based on his best performer, as defined in the following equation.

30 $$\:\left\{\begin{array}{c}{v}_{Tfi}(t+1)=\left(p\times\:{x}_{Tfi}\left(t\right)+\left(1-p\right)\times\:{x}_{Tf}\left(t\right)+R\times\:\left({x}_{Tf}\left(t\right)-{x}_{Tfi}\left(t\right)+p\times\:\left(\overline{{x}_{T}}\left(t\right)-\overline{{x}_{K}}\left(t\right)\right)\right)\right)\times\:\alpha\:+\beta\:\\\:Tfi=Tfi+1\end{array}\right.$$

Here, $\:Tfi$ is the index of Tianji’s current fastest horse, and $\:{x}_{Tf}$​ represents the best horse in his group. In Eq. (30), $\:{x}_{Tfi}$ is updated toward $\:{x}_{Tf}$​, considering the performance gap between the two populations and the strength of $\:{x}_{Tf}$​. To respond, the king’s current fastest horse is replaced with reference to Tianji’s top performer, as defined below:

31 $$\:\left\{\begin{array}{c}{v}_{Kfi}(t+1)=\left(p\times\:{x}_{Kfi}\left(t\right)+\left(1-p\right)\times\:{x}_{Tfi}\left(t\right)+R\times\:\left({x}_{Tfi}\left(t\right)-{x}_{Kfi}\left(t\right)+p\times\:\left(\overline{{x}_{T}}\left(t\right)-\overline{{x}_{K}}\left(t\right)\right)\right)\right)\times\:\alpha\:+\beta\:\\\:Kfi=Kfi+1\end{array}\right.$$

**Fig. 7:**
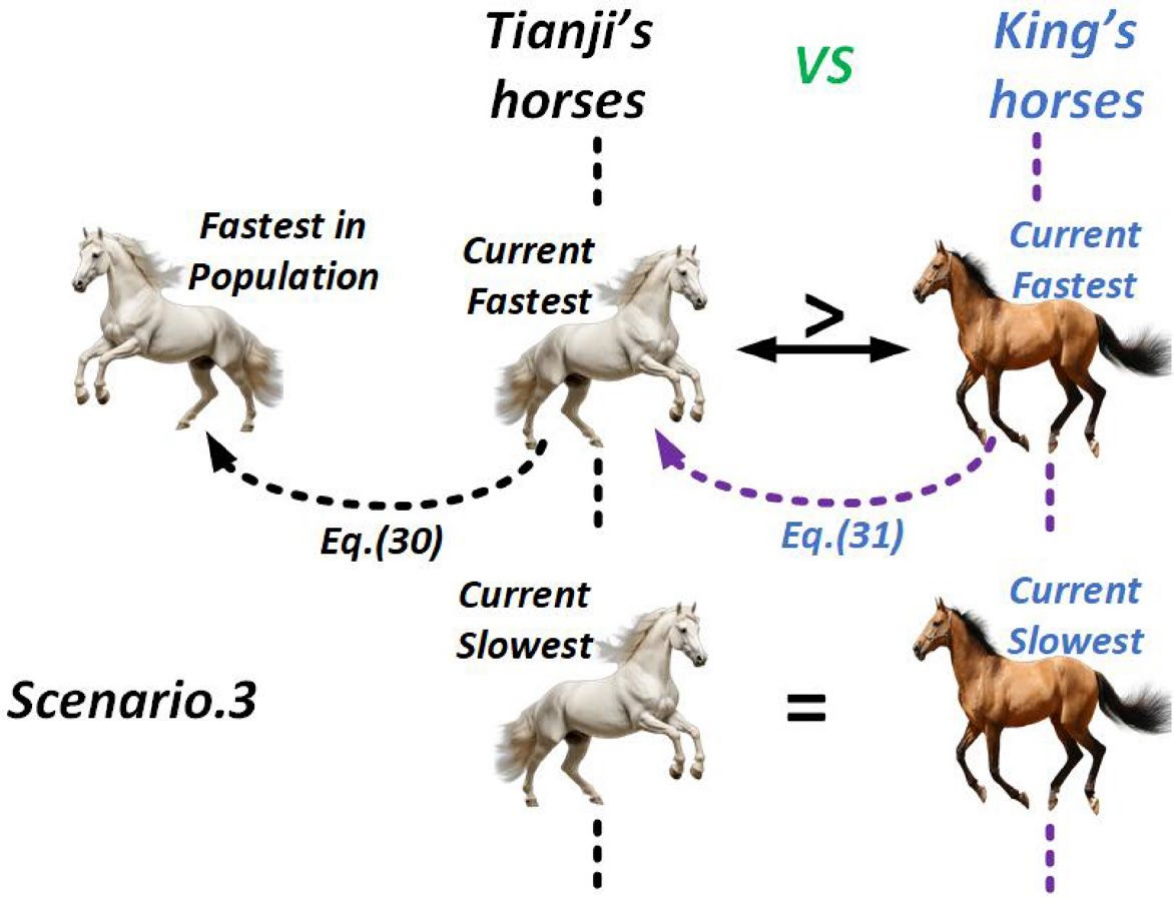
THRO Competition Approach in Scenario 3.

Figure 7 illustrates the THRO competition strategy for Scenario 3, where the symbol “=” signifies that the both sides horses are running at equal speed.

##### Scenario 4

When Tianji’s slowest horse equals the king’s slowest but his fastest is weaker than the king’s, he deliberately pits the slowest against the king’s strongest, sacrificing it to neutralize the opponent’s best. Since the loss is unavoidable, the algorithm updates Tianji’s slowest horse using a randomly selected one from his population. The replacement is defined as.

32 $$\:\left\{\begin{array}{c}{v}_{Tsi}(t+1)=\left(p\times\:{x}_{Tsi}\left(t\right)+\left(1-p\right)\times\:{x}_{Tr2}\left(t\right)+R\times\:\left({x}_{Tr2}\left(t\right)-{x}_{Tsi}\left(t\right)+p\times\:\left(\overline{{x}_{T}}\left(t\right)-\overline{{x}_{K}}\left(t\right)\right)\right)\right)\times\:\alpha\:+\beta\:\\\:Tsi=Tsi-1\end{array}\right.$$

Let $\:{x}_{Tr2}$​ be a randomly chosen horse from Tianji’s population. In Eq. (32), $\:{x}_{Tsi}$​ is updated toward $\:{x}_{Tr2}$​, considering both the population strength gap and $\:{x}_{Tr2}$​’s performance. Similarly, the king updates his current fastest horse using his top performer, as defined by:

33 $$\:\left\{\begin{array}{c}{v}_{Kfi}(t+1)=\left(p\times\:{x}_{Kfi}\left(t\right)+\left(1-p\right)\times\:{x}_{Kfi}\left(t\right)+R\times\:\left({x}_{Kf}\left(t\right)-{x}_{Kfi}\left(t\right)+p\times\:\left(\overline{{x}_{T}}\left(t\right)-\overline{{x}_{K}}\left(t\right)\right)\right)\right)\times\:\alpha\:+\beta\:\\\:Kfi=Kfi+1\end{array}\right.$$

**Fig. 8:**
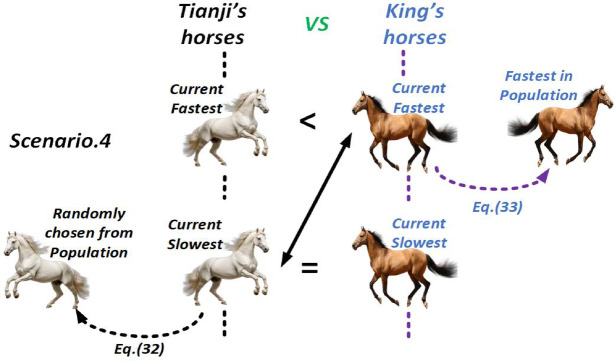
THRO Competition Approach in Scenario 4.

Figure 8 illustrates the THRO algorithm’s competition strategy in scenario 4.

##### Scenario 5

When Tianji’s slowest horse matches the king’s slowest, and his fastest horse equals the king’s fastest, he pits the slowest horse against the king’s fastest, accepting a loss. The algorithm then applies the same update strategy as in Scenario 4, with the update for Tianji’s slowest horse defined as.

34 $$\:\left\{\begin{array}{c}{v}_{Tsi}(t+1)=\left(p\times\:{x}_{Tsi}\left(t\right)+\left(1-p\right)\times\:{x}_{Tr3}\left(t\right)+R\times\:\left({x}_{Tr3}\left(t\right)-{x}_{Tsi}\left(t\right)+p\times\:\left(\overline{{x}_{T}}\left(t\right)-\overline{{x}_{K}}\left(t\right)\right)\right)\right)\times\:\alpha\:+\beta\:\\\:Tsi=Tsi-1\end{array}\right.$$

Here, $\:{x}_{Tr3}$​ denotes any horse randomly taken from Tianji’s collection. The king’s current fastest one is replaced as follows:

35 $$\:\left\{\begin{array}{c}{v}_{Kfi}(t+1)=\left(p\times\:{x}_{Kfi}\left(t\right)+\left(1-p\right)\times\:{x}_{Kf}\left(t\right)+R\times\:\left({x}_{Kf}\left(t\right)-{x}_{Kfi}\left(t\right)+p\times\:\left(\overline{{x}_{T}}\left(t\right)-\overline{{x}_{K}}\left(t\right)\right)\right)\right)\times\:\alpha\:+\beta\:\\\:Kfi=Kfi+1\end{array}\right.$$

**Fig. 9:**
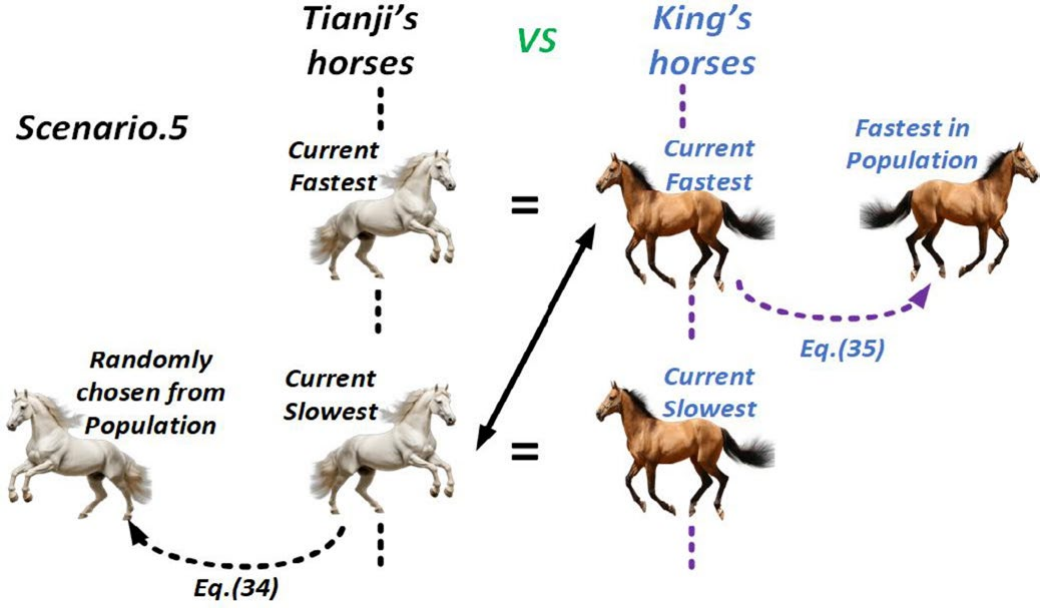
THRO Competition Approach in Scenario 5.

Figure 9 illustrates the THRO algorithm’s competition strategy for scenario 5.

As in Tianji’s horse racing tale, the strategy’s success relies on the king’s horses being slightly superior. In the THRO algorithm, this is captured by the term $\:p\times\:\left({x}_{T}\left(t\right)-{x}_{k}\left(t\right)\right)$, representing the average quality gap between populations. This gap guides Tianji’s strategy, producing diverse search behaviors that enhance the algorithm’s global optimization performance.

#### Training

Since THRO is an iterative search process, reinforcing solutions from previous iterations is crucial. To avoid stagnation, a training strategy is applied: after each round, horses from both populations undergo performance enhancement through interactions with horses of different speeds and focused training with the fastest horse. This strategy is formulated as:

36 $$\:{v}_{Ti}^{j}(t+1)=\left\{\begin{array}{c}{x}_{Ti}^{j}\left(t\right)+{L}_{T}\times\:\left({x}_{Tr4}^{j}-{x}_{Tr5}^{j}\right)\:\:\:\:\:\:\:\:\:\:\:if\:rand<0.5\\\:{x}_{Tf}^{j}\left(t\right)+{M}_{T}\times\:\left({x}_{Tf}^{j}\left(t\right)-{x}_{Ti}^{j}\left(t\right)\right)\:\:\:\:\:\:\:\:\:\:\:\:\:\:\:\:\:\:\:\:else\end{array}\right.$$

37 $$\:\left\{\begin{array}{c}{L}_{T}=0.2\times\:L\\\:{M}_{T}=0.5\times\:\left(1+0.001\times\:{\left(1-\frac{t}{T}\right)}^{2}\times\:\text{si}\text{n}\left(\pi\:\times\:rand\right)\right)\end{array}\right.$$

38 $$\:{v}_{Ki}^{j}(t+1)=\left\{\begin{array}{c}{x}_{Ki}^{j}\left(t\right)+{L}_{K}\times\:\left({x}_{Kr1}^{j}-{x}_{Kr2}^{j}\right)\:\:\:\:\:\:\:\:\:\:\:if\:rand<0.5\\\:{x}_{Kf}^{j}\left(t\right)+{M}_{K}\times\:\left({x}_{Kf}^{j}\left(t\right)-{x}_{Ki}^{j}\left(t\right)\right)\:\:\:\:\:\:\:\:\:\:\:\:\:\:\:\:\:\:\:\:else\end{array}\right.$$

39 $$\:\left\{\begin{array}{c}{L}_{K}=0.2\times\:L\\\:{M}_{K}=0.5\times\:\left(1+0.001\times\:{\left(1-\frac{t}{T}\right)}^{2}\times\:\text{sin}\left(\pi\:\times\:rand\right)\right)\end{array}\right.$$

Let $\:{x}_{Ti}^{j}$and $\:{x}_{Ki}^{j}$​ denote the $\:j-th$ attribute of the $\:i\:-th$ horse in Tianji’s and the king’s populations, respectively. $\:{x}_{Tf}^{j}$​ and $\:{x}_{Kf}^{j}$ represent the fastest horse in each lineup. Indices $\:Tr4$ and $\:Tr5$ (Tianji) and $\:Kr1$ and $\:Kr2$ (king) indicate randomly selected horses. $\:T$ is the maximum number of iterations, while $\:{L}_{T}$ and $\:{M}_{T}$​ and $\:{L}_{K}$ and $\:{M}_{K}$​ are the training parameters for Tianji’s and the king’s horses, respectively.

#### THRO algorithm procedure

The Tianji’s Horse Racing Optimization (THRO) algorithm is inspired by Tianji’s strategic horse races, where adaptive decisions turn disadvantages into strengths. Two populations Tianji’s and the king’s represent candidate solutions, with horses ranked from fastest to slowest (ascending fitness for minimization). Each iteration includes n racing rounds, with updates applied according to the relative performance of the slowest and fastest horses. Updates consider references to the fastest, slowest, or randomly selected horses, as well as the overall quality gap between populations, balancing exploration and exploitation.

**Fig. 10:**
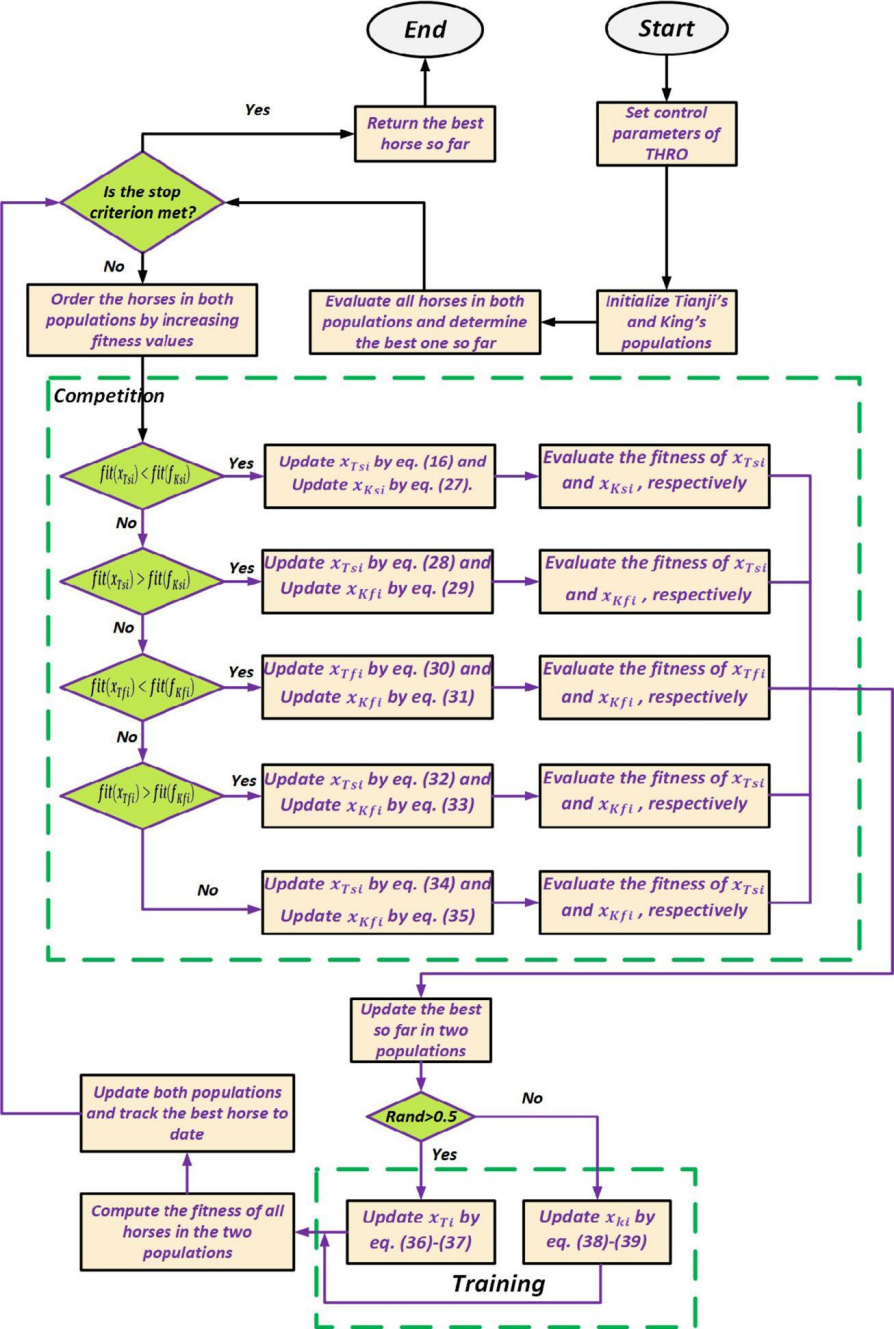
Flowchart of the THRO.

Weaker horses may be deliberately sacrificed to offset stronger opponents, and candidate solutions are reinforced through a training phase involving interactions with other horses and the top performer. Mutation and Lévy flights allow both short adjustments and long jumps, preventing stagnation. Horses are replaced only if candidates improve performance. Iterations continue until the stopping criterion is met, and the best-performing horse is returned as the optimal solution. THRO flowchart is presented in Fig. 10.

#### Computational complexity analysis

Analyzing the computational complexity of the THRO algorithm is essential to evaluate its performance, as it depends on key parameters: population size $\:\varvec{n}$ of Tianji’s and the king’s horses, problem dimensionality $\:\varvec{d}$, and maximum iterations $\:\varvec{T}$. Considering these factors, the total cost of THRO can be expressed as:

40 $$\begin{aligned} \:O\left(THRO\right) = & \; O\left(Problem\:Definition\right)+O\left(Initialization\right)+O\left(Function\:Evaluation\right) \\ & +O\left(Tianji's\:Horse\:Updates\:in\:Competition\right)+O\left(King's\:Horse\:Updates\:in\:Competition\right) \\ & +\:O\left(Tianji's\:Horse\:Updates\:in\:Training\right)+O\left(King's\:Horse\:Updates\:in\:Training\right) \\ & +\:O\left(Sorting\:Tianji's\:Horses\right)+O\left(Sorting\:King's\:Horses\right)\end{aligned}$$

Breaking this down:

41 $$\:=O\left(1\right)+O\left(2n\right)+O\left(2Tn\right)+5O\left(\frac{1}{5}Tnd\right)+5O\left(\frac{1}{5}Tnd\right)+O\left(Tnd\right)+O\left(Tnd\right)+O(Tnlogn)+O(Tnlogn)\:$$

This simplifies to:

42 $$\:O(2Tnlogn+4Tnd+2Tn+2n+1)\approx\:O\left(Tn\right(d+logn\left)\right)\:$$

This shows that THRO’s complexity grows with problem size, dimensionality, and iteration count.

For comparison, PSO, ABC, and HHO have a complexity of $\:O\left(Tn\right(d+logn\left)\right)$^79–81^, as they mainly involve population updates and fitness evaluation. SMA and THRO include additional sorting and comparison operations, adding the $\:logn$ term, making them more suitable for structured optimization problems. Simpler algorithms like PSO, ABC, and HHO are better for dynamic problems requiring fast adaptation.

In summary, computational complexity reflects an algorithm’s strategy and helps guide the choice of the most suitable method for a given optimization problem.

## The specific design layout of the proposed PDA-FOPIID controller

This section outlines the framework of the presented PDA-FOPIID controller. The PDA-FOPIID controller combines the strengths of both traditional and fractional-order control strategies to offer enhanced dynamic performance and robustness. The P (Proportional) term improves the speed of response by providing immediate correction based on the current error. The D (Derivative) term adds anticipatory action, helping to dampen oscillations and reduce overshoot. The A (Acceleration) term, introduced as an additional second-order derivative component, enhances system responsiveness, particularly in rapidly changing or dynamic environments allowing the controller to react more effectively to the rate of change of acceleration (jerk) and further stabilize the system.

**Fig. 11:**
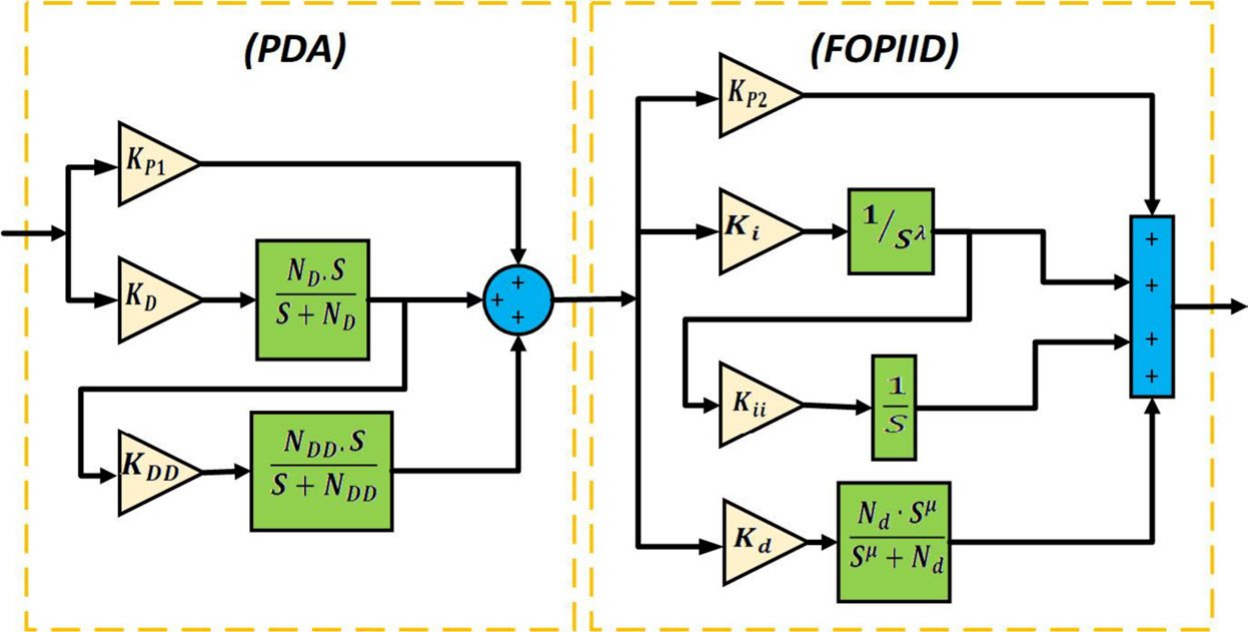
Control scheme of the proposed system.

The FOPIID part contributes significant flexibility through fractional calculus. The FO-I (Fractional-Order Integral) term improves steady-state accuracy while avoiding the sluggishness often caused by traditional integral action. The FOD (Fractional-Order Derivative) allows for a more refined damping effect than integer-order D, enhancing both noise rejection and transient performance. Together, the two fractional orders provide tunable memory effects, enabling the controller to better adapt to the specific dynamics of complex or nonlinear systems. This hybrid structure makes the PDA-FOPIID controller highly suitable for Interconnected systems requiring fast response, precision, and robustness. Figure 11 illustrates the architecture of the PDA-FOPIID regulator, and this can be described mathematically as follows:

43 $$\:H\left(s\right)=\:C1\text{}\left(s\right)\cdot\:C2\text{}\left(s\right)$$

44 $$\:{C}_{1}\text{}\left(\text{s}\right)={K}_{P}+{K}_{D}.\left(\frac{{N}_{D}.\:\:S}{S+{N}_{D}}\right)+{K}_{D}.\left(\frac{{N}_{D}.\:\:S}{S+{N}_{D}}\right).{K}_{DD}.\left(\frac{{N}_{DD}.\:\:S}{S+{N}_{DD}}\right)$$

45 $$\:{C}_{2}\left(s\right)={K}_{p}+{K}_{i}\frac{1}{{S}^{\lambda\:}}+{K}_{i}\frac{1}{{S}^{\lambda\:}} \cdot {K}_{ii}.\frac{1}{S}+{K}_{d}.\left(\frac{{N}_{d}.\:{\:S}^{\mu\:}}{{S}^{\mu\:}+{N}_{d}}\right)$$

Ultimately, the optimal values of $\:{K}_{P1},\:{K}_{D},\:{N}_{D},\:{K}_{DD},\:{N}_{DD},\:{K}_{P2},\:{K}_{i},\:\lambda\:,\:{K}_{ii},\:{K}_{d},\:{N}_{d}$ and $\:\mu\:$ for the proposed PDA-FOPIID controllers are obtained through the THRO algorithm. The algorithm is employed to minimize the fitness function -Integral of Time Squared Error (ITSE)- as defined in Eq. (44).

46 $$\:J=ITSE={\int\:}_{0}^{t}{t\left[\right(\varDelta\:{f}_{1})}^{2}{+{\left(\varDelta\:{f}_{2}\right)}^{2}+\left(\varDelta\:{P}_{tieline}\right)}^{2}+{\left(\varDelta\:{V}_{1}\right)}^{2}{+\left(\varDelta\:{V}_{2}\right)}^{2}]dt$$

These parameter ranges are determined in the algorithm’s code using two arrays: the lower bound array and the upper bound array, which define the lower and upper allowable values for each parameter, respectively, as shown in next equation. The minimum values can be determined as lb = [0, 0, 100, 0, 100, 0, 0, 0, 0, 0, 100, 0], while the upper bounds are given by ub = [2, 2, 500, 0.1, 500, 2, 2, 1, 0.1, 2, 500, 1].

47 $$\:\left\{\begin{array}{c}\begin{array}{c}{K}_{P1}min\:<\:{K}_{P1}\:<\:{K}_{P1}max\\\:{K}_{D}min\:<\:{K}_{D}\:<\:{K}_{D}max\\\:{N}_{D}min\:<\:{N}_{D}\:<\:{N}_{D}max\end{array}\\\:\begin{array}{c}{K}_{DD}min\:<\:{K}_{DD}\:<\:{K}_{DD}max\\\:\begin{array}{c}{N}_{DD}min\:<\:{N}_{DD}\:<\:{N}_{DD}max\\\:\begin{array}{c}{K}_{P2}min\:<\:{K}_{P2}\:<\:{K}_{P2}max\\\:{K}_{i}min\:<\:{K}_{i}\:<\:{K}_{i}max\end{array}\\\:\lambda\:min\:<\:\lambda\:\:<\:\lambda\:max\end{array}\\\:{K}_{ii}min\:<\:{K}_{ii}\:<\:{K}_{ii}max\end{array}\\\:\begin{array}{c}{K}_{d}min\:<\:{K}_{d}\:<\:{K}_{d}max\\\:{N}_{d}min\:<\:{N}_{d}\:<\:{N}_{d}max\\\:\mu\:min\:<\:\mu\:\:<\:\mu\:max\end{array}\end{array}\right.$$

Therefore, this study aims to minimize the Integral of Time Squared Error (ITSE) by employing the THRO strategy to configure the optimal factors of the presented regulator. Following optimization, the controller’s performance is assessed and compared with other controllers based on key time-domain metrics, including maximum overshoot (MO), maximum undershoot (MU), settling time (ST), and rise time (RT).

## Analysis of simulation results

This phase evaluates the dynamic performance of a dual-area networked hybrid system controlled by the suggested PDA-FOPIID controller, which is optimized using the THRO algorithm.

The superior performance of the proposed PDA–FOPIID controller can be understood through control theory insights. The acceleration term in the PDA stage improves damping and accelerates transient response compared with classical PID and PD-based schemes. The fractional-order structure of the FOPIID stage provides additional tuning flexibility, enabling a better trade-off between robustness, steady-state accuracy, and dynamic performance than integer-order controllers. Furthermore, the cascaded arrangement facilitates a natural decoupling of voltage and frequency regulation, mitigating the adverse effects of LFC–AVR coupling. In combination with precise parameter tuning via Tianji’s Horse Racing Optimization, these features allow the PDA-FOPIID controller to achieve superior stability margins and the lowest error indices compared with other benchmark strategies.

The assessment is carried out in two primary phases: First, the THRO optimization algorithm is benchmarked verses several well-established approaches namely GTO, PSO, WHO, and GBO With respect to productivity and effectiveness. The benchmarking process involves 200 iterations with size of population of 30, under a sudden load disturbance applied at $\:t\:=\:20$ s in Area A. The THRO algorithm demonstrates superior fitness performance, achieving an average improvement of approximately $\:45\%$ over the other methods. Second, the reliability and stability of the presented THRO-optimized PDA-FOPIID controller are compared with other THRO-tuned controllers, including TID, PID, FOPID, PD-(1 + PI), and FOPI-PIDD². This comparative evaluation is conducted under a variety of operating scenarios, such as sudden disturbances in the load (SLD), multi-step disturbances (MSD), random load variations, integration of renewable energy sources (RESs), and high-RES penetration levels. The analysis focuses on each controller’s ability to effectively regulate system frequency, deviations in tie-line power (ΔPtie) and terminal voltage. These two evaluation phases are thoroughly detailed in the following segments.

### Assessment of THRO algorithm performance

**Fig. 12:**
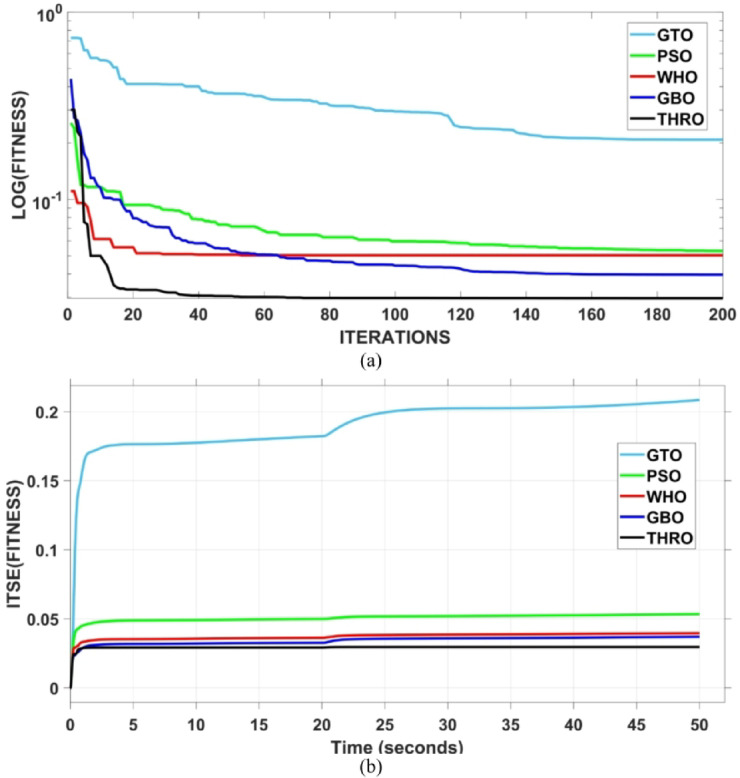
Best fitness for the compared five algorithms-(a) vs. iterations (b) vs. time.

This subsection presents an evaluation of the THRO algorithm’s effectiveness in load frequency control (LFC) and automatic voltage regulation (AVR) applications. The performance of THRO is benchmarked against several well-established optimization algorithms from the literature, including GTO, PSO, WHO, and GBO. The THRO algorithm demonstrates superior efficiency, achieving significant improvements in fitness value approximately$\:\:86\text{\%}$ over GTO, $\:44\%$ over PSO, $\:30\%$ over WHO, and $\:23\%$ over GBO. This comparative analysis focuses on assessing each algorithm’s ability to well tune the factors of the presented PDA-FOPIID controller and maintain voltage and frequency stability in the interconnected two-area hybrid power system. Figure 12 (a and b) depicts the convergence behavior of all algorithms over 200 iterations, presenting the best cost versus iteration curves and highlighting the speed at which the selected algorithm attains the optimal value. Furthermore, Table 1 summarizes the optimal controller parameters for the PDA-FOPIID controller as obtained from each of the tuning approaches under study: GTO, PSO, WHO, GBO, and THRO.

**Table 1:** Optimum settings of the proposed (PDA-FOPIID) controller optimized through the five approaches.

	Parameters	GTO	PSO	WHO	GBO	THRO
LFC_a_	K_P1_	1	1.996	2	1.999	1.999
	K_D_	1	1.926	1.999	1.993	2
	N_D_	101	266	100	101	498
	K_DD_	0	0.047	0	$\:0$	$\:0$
	N_DD_	101	248	100	245	100
	K_P2_	1	1.882	1.999	1.997	1.937
	K_i_	1	1.997	1.999	1.999	1.999
	$\lambda$	0.710	0.932	0.941	0.999	0.993
	K_ii_	0.083	0.040	0.1	0.021	0.020
	K_d_	1	1.041	0	0.004	1.992
	N_d_	162	272	101	472	500
	$\mu$	0	0.005	1	0.472	0
AVR_a_	K_P1_	1	1.114	2	1.998	1.994
	K_D_	0.152	1.505	0.103	0.065	0.102
	N_D_	101	299	500	500	500
	K_DD_	0	0.099	0.036	0.034	0.039
	N_DD_	100	500	500	496	500
	K_P2_	1	0.924	2	1.997	1.999
	K_i_	0.709	2	2	1.999	1.999
	$\lambda$	0.756	0.528	0.355	0.184	0.246
	K_ii_	0.012	0.029	0.044	0.099	0.074
	K_d_	0.999	0.386	1.534	1.033	1.349
	N_d_	101	259	431	500	374
	$\mu$	0.212	0.790	0.681	0.832	0.713
LFC_b_	K_P1_	1	1.995	2	1.992	1.981
	K_D_	0.005	0.014	0	0	0
	N_D_	101	316	171	105	480
	K_DD_	0	0.007	0	0.083	0
	N_DD_	101	356	285	500	103
	K_P2_	1	2	1.994	1.999	1.362
	K_i_	0.999	1.960	2	1.999	1.997
	$\lambda$	0.515	0.937	1	0.987	0.944
	K_ii_	0	0.011	0	0	0.001
	K_d_	0	1.918	2	1.999	1.999
	N_d_	100	257	100	100	335
	$\mu$	0.507	0	0	0	0.008
AVR_b_	K_P1_	1	1.799	2	1.999	1.999
	K_D_	0.999	0.097	0.106	0	0.100
	N_D_	100	488	500	415	500
	K_DD_	0	0.099	0.1	0.099	0.049
	N_DD_	500	288	500	488	486
	K_P2_	0	1.981	1.999	1.998	1.999
	K_i_	1	1.997	2	1.999	1.999
	$\lambda$	0.598	0.467	0.414	0.196	0.398
	K_ii_	0.019	0.029	0.036	0.1	0.036
	K_d_	0.183	1.999	1.944	0.838	1.678
	N_d_	499	294	497	499	419
	$\mu$	0	0.702	0.715	0.943	0.672
ITSE		0.208	0.053	0.042	0.038	0.029

### Evaluation of the effectiveness of the proposed PDA-FOPIID controller

To assess the effectiveness of the suggested controller in enhancing the efficiency of a two-area microgrid, a comprehensive set of simulations was conducted via the MATLAB/Simulink. These simulations were strategically established to examine the controller’s robustness and efficiency under a variety of operating cases and disturbances. The analyses are organized into the following segments, each representing a distinct test case:

- **Scenario I**: At $\:t\:=\:20$ seconds, a 1% step disturbance in load is imposed on Area A.
- **Scenario II**: A multi-step disturbance (MSD) is applied.
- **Scenario III**: A load disturbance is randomly subjected to Area A.
- **Scenario IV**: Connection of renewable energy sources (RESs) is simulated.
- **Scenario V**: A high penetration of RESs is examined.

A. **Scenario I: At t = 20 s**,** a 1% step disturbance in load is imposed on Area A**.

In the initial test scenario, a step disturbance of the load (SLD) is subjected to Area A at $\:t\:=\:20$ seconds to assess the performance of the proposed TDA-FOPIID controller and its collaboration with the electric vehicle (EV) system. The main objective is to evaluate the system’s resilience in maintaining voltage and frequency stability under perturbations. Simulation results confirm that both the Load Frequency Control (LFC) and Automatic Voltage Regulation (AVR) systems show outstanding capability when using the proposed PDA-FOPIID controller optimized by Tianji’s horse racing optimization (THRO) algorithm.

**Fig. 13:**
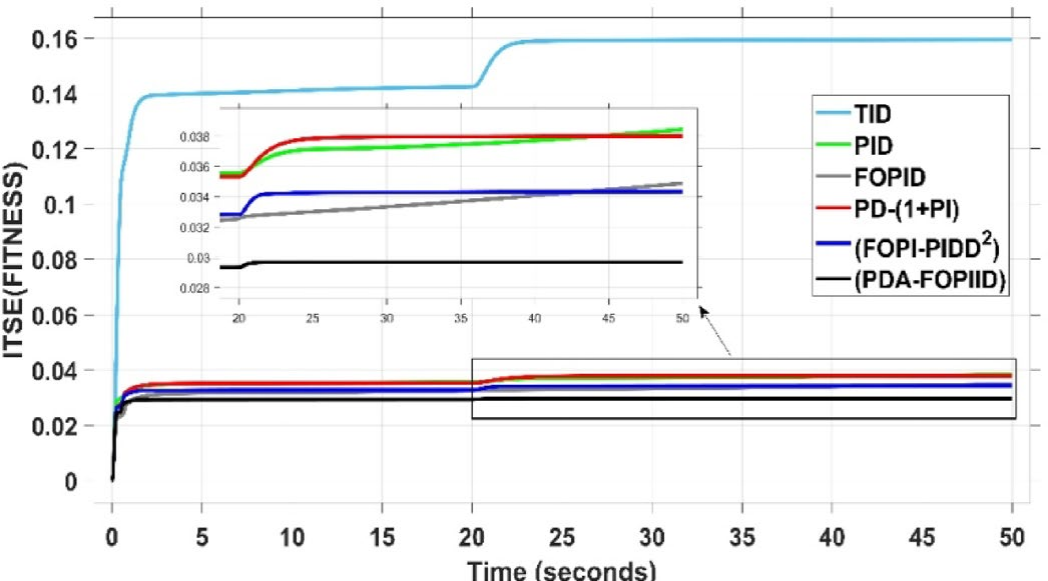
Best cost versus time in Case I.

**Fig. 14:**
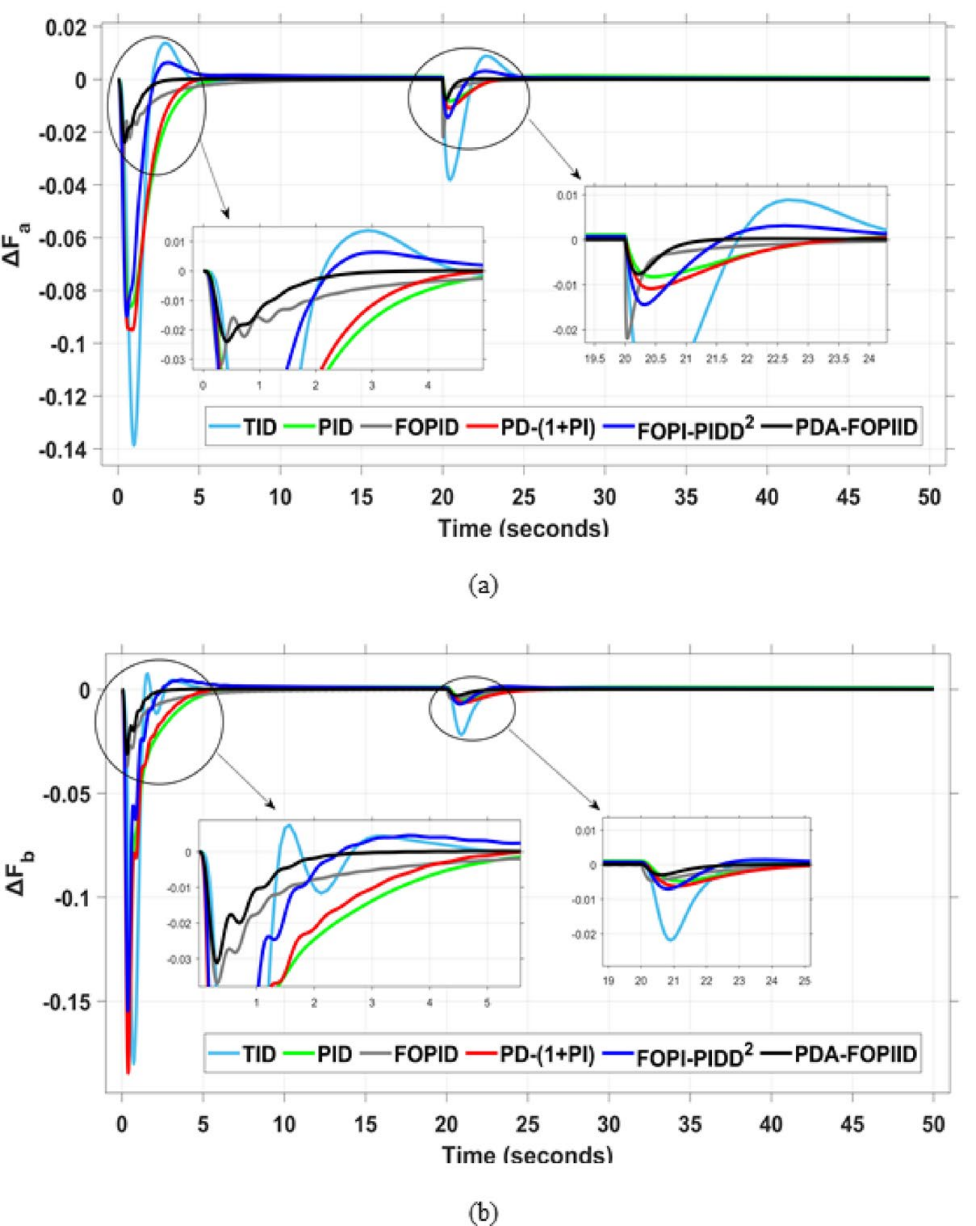
Frequency curves based on Case I characteristics- (**a**) ΔF_a_ (**b**) ΔF_b_.

**Fig. 15:**
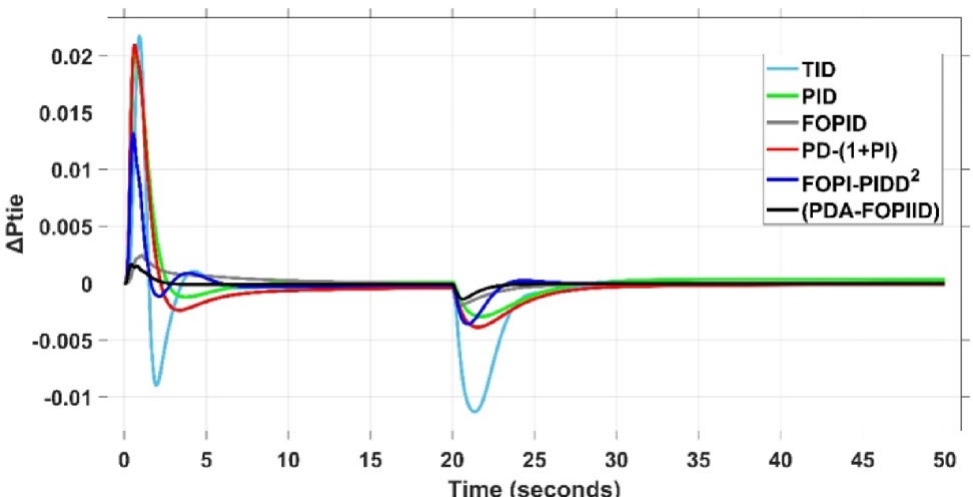
ΔPtie curves based on Case I characteristics.

**Fig. 16:**
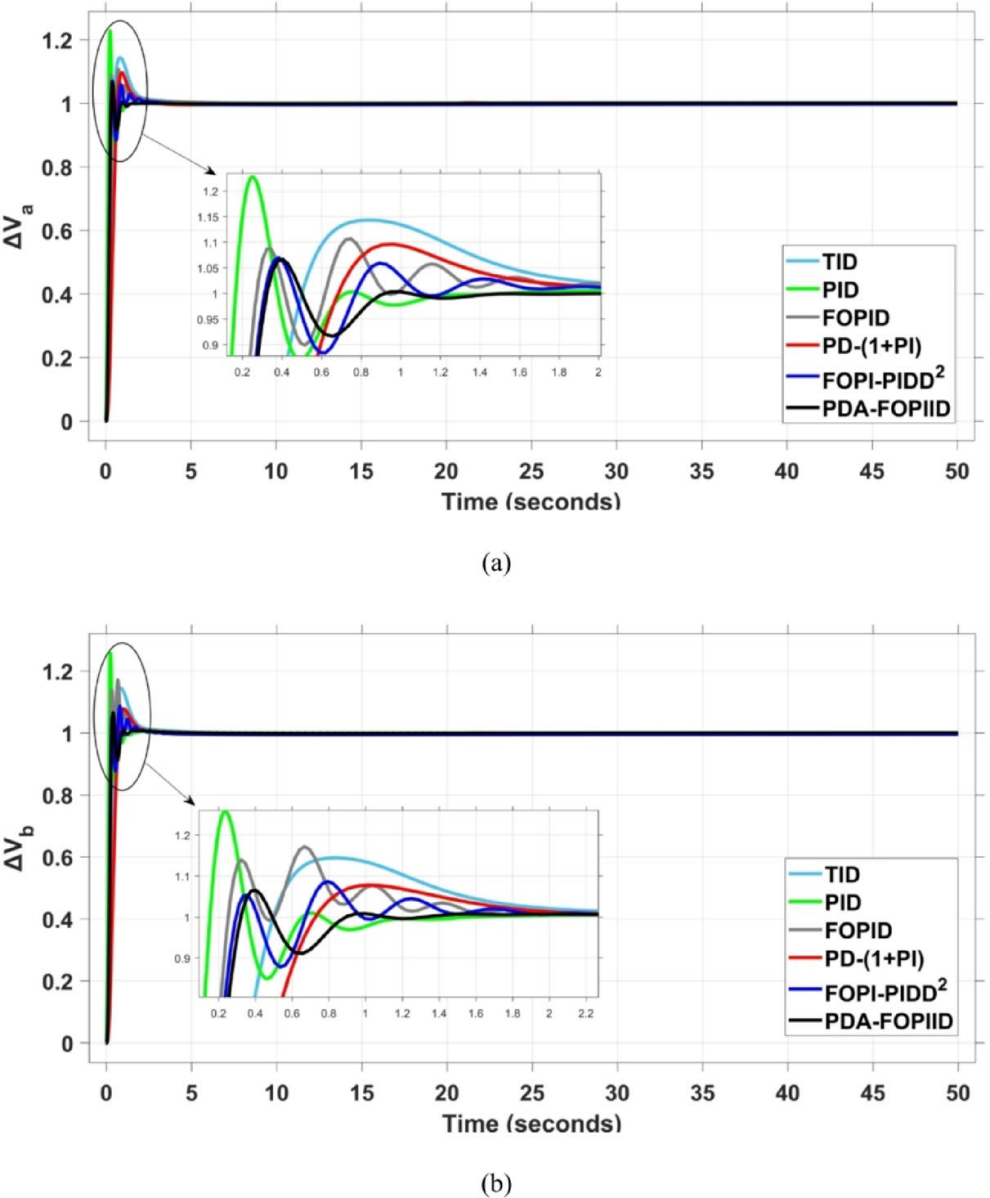
Voltage curves based on Scenario I characteristics- (**a**) ΔV_a_, (**b**) ΔV_b_.

When compared to other THRO -tuned controllers namely PID, FOPID, TID, FOPI-PIDD² and PD-(1 + PI) the proposed controller achieves notably reduced undershoot, overshoot, and settling time. As shown in Fig. 13, the PDA-FOPIID controller demonstrates the best fitness value of $\:0.0297$, outperforming all the compared controllers. Detailed fitness values for each controller are presented in Table 2. Figure 14 highlights that the presented regulator reaches to the minimum value of maximum overshoot (MOS), maximum undershoot (MUS), and settling time (ST) for frequency deviations (ΔFₐ and ΔF_b_) at both the simulation start and during the SLD. Specifically, for ΔFₐ, the MOS is 0, MUS is $\:-0.0237$, and ST is $\:2.66\:s$, for ΔF_b_, the MOS is $\:0$, MUS is $\:-0.0311$, and ST is $\:2.72\:s$. Figure 15 illustrates that for tie-line power deviation, the presented controller achieves the lowest MOS ($\:1.66\:\times\:\:{10}^{-3}$), MUS ($\:-1.37\:\times\:\:{10}^{-3 3}$), and a settling time of $\:3\:s$, demonstrating superior damping characteristics and dynamic response. Figure 16 shows the performance of terminal voltage deviation (ΔV_a_ and ΔV_b_), where the proposed controller again provides the best performance, with MOS values of $\:1.066$ and $\:1.065$, settling times of $\:1.5\:s$ and $\:1.8\:s$, and rise times (RT) of $\:0.325\:s$ for both areas. The performance outcomes, demonstrating the strength of the presented regulator, are summarized in Table 3.

Overall, when compared to PD-(1 + PI), FOPI-PIDD², TID, FOPID, and PID controllers, the proposed PDA-FOPIID regulator improves performance by $\:54\%$, $\:59\%$, $\:62\%$, $\:33\%$, and $\:32\%$, respectively. These improvements are achieved by minimizing the fitness function to its lowest value of $\:0.0297$, confirming the proposed PDA-FOPIID controller tuned via THRO as a highly effective and reliable solution for dual-area hybrid system LFC and AVR applications.

**Table 2:** Fitness values for compared controller with scenario I.

Controllers	Fitness				
	ΔF_a_	ΔF_b_	ΔV_a_	ΔV_b_	Total
TID	0.02029	0.01623	0.061	0.06193	0.15945
PID	0.002779	0.0164	0.003122	0.01613	0.038429
FOPID	0.000237	0.0165	0.000249	0.01789	0.034876
PD-(1 + PI)	0.003336	0.01568	0.00336	0.01562	0.037997
FOPI-PIDD^2^	0.001974	0.01564	0.001329	0.01538	0.034327
PDA-FOPIID (Proposed)	0.000433	0.0002583	0.01476	0.01423	0.0296815

**Table 3:** The dynamic characteristics of the compared controllers with scenario I.

Controllers		TID	PID	FOPID	PD-(1 + PI)	FOPI-PIDD^2^	PDA-FOPIID (Proposed)
ΔF_a_	**MOS**	0.0137	0	0	0	0.00631	0
	**MUS**	−0.1379	−0.086	−0.0317	−0.0948	−0.0896	−0.0237
	**ST**	7.22	7.09	13	14	12.6	2.66
ΔF_b_	**MOS**	0.0075	0	0	0	0.0046	0
	**MUS**	−0.18	−0.167	−0.0367	−0.1845	−0.1545	−0.0311
	**ST**	6	8	16	12	15	2.72
ΔPtie	**MOS** $\:\times\:{10}^{-3}$	021.7	020.3	2.4	20.8	12.9	1.66
	**MUS** $\:\:\times\:{10}^{-3}$	−11.3	−2.9	−1.9	−3.8	−3.5	−1.37
	**ST**	8	12	15	13	7	3
ΔV_a_	**MOS**	1.143	1.226	1.10067	1.095	1.07	1.066
	**MUS**	0.5	0.163	0.28	0.708	0.311	0.325
	**ST**	8	9	10	6	5	1.5
ΔV_b_	**MOS**	1.144	1.252	1.172	1.077	1.086	1.065
	**RT**	0.49	0.15	0.247	0.751	0.31	0.325
	**ST**	2.75	2	2.5	2.5	2.7	1.8

B. **Scenario II: A multi-step disturbance (MSD) is applied.**

**Fig. 17:**
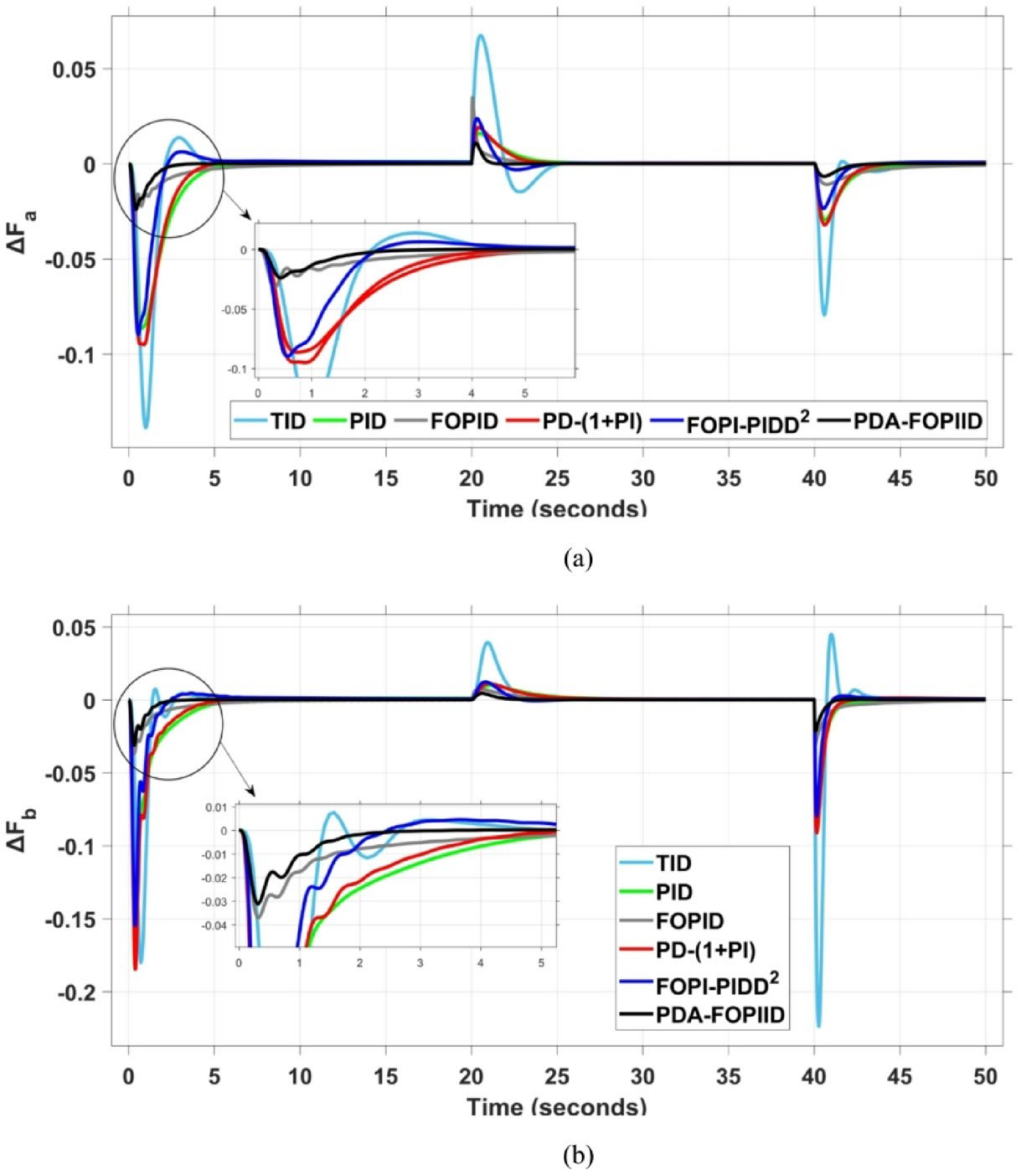
Frequency curves based on case II characteristics- (**a**) ΔF_a_, (**b**) ΔF_b_.

**Fig. 18:**
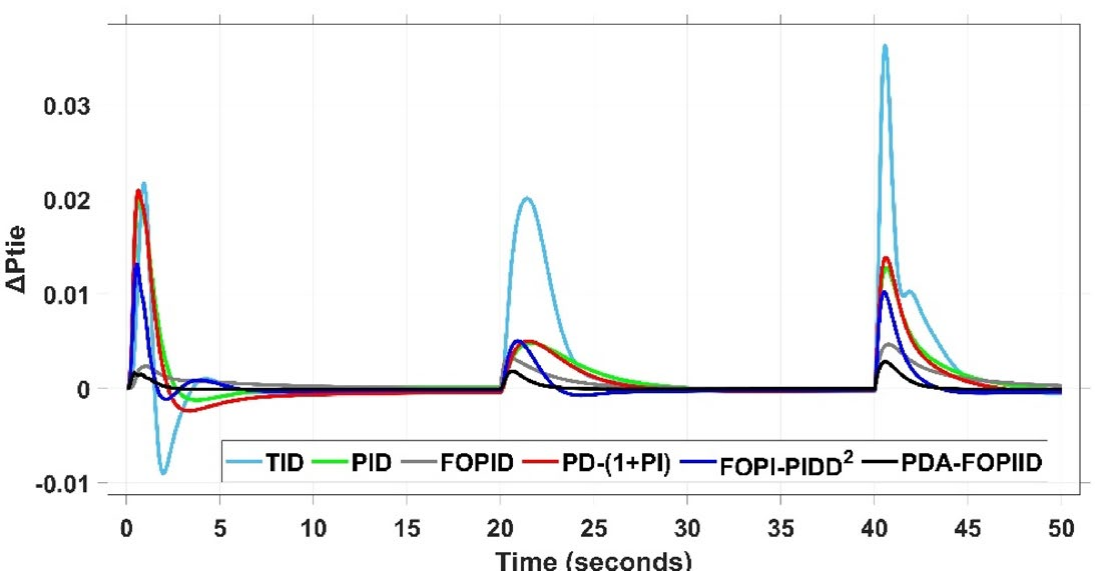
ΔPtie curves based on case II.

**Fig. 19:**
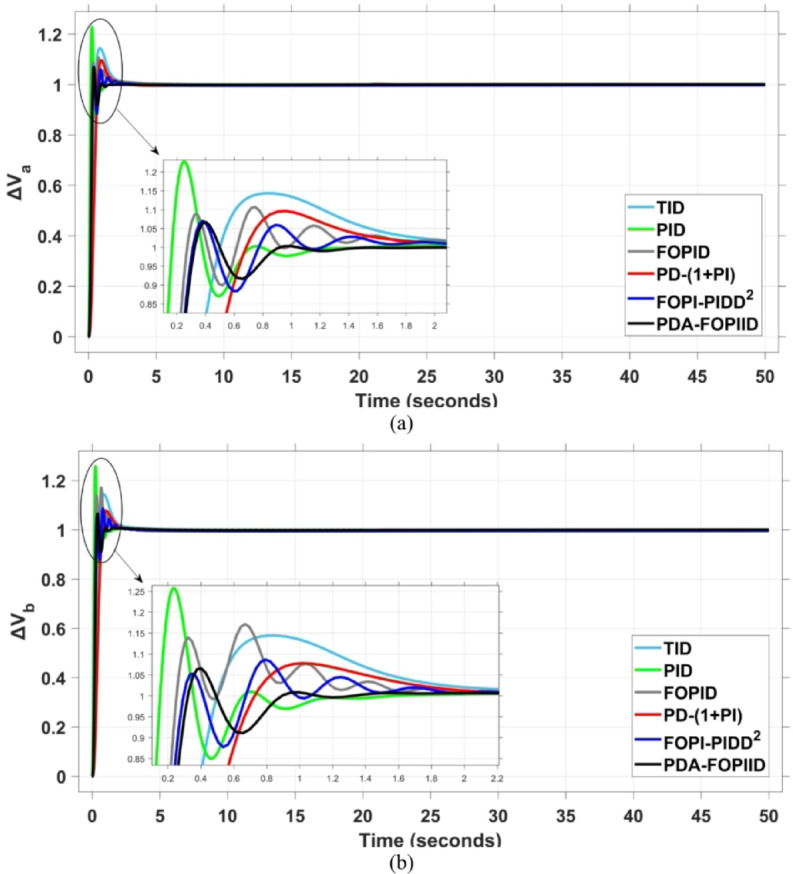
Voltage response based on case II characteristics- (**a**) ΔV_a_, (**b**) ΔV_b_.

This segment analyzes the system’s dynamic behavior in case of a multi-step disturbance (MSD) applied to both areas of the interconnected system. Specifically, Area A is subjected to a disturbance of $\:-0.3\:pu$ at $\:t\:=\:20$ seconds, followed by a second disturbance of $\:0.3\:pu$ in Area B at $\:t\:=\:40$ seconds. The metric curves corresponding to the six tested regulators are presented in Figs. 17 and 18, and 19. These figures consistently highlight the superior performance of the presented PDA-FOPIID regulator, which has been optimized using Tianji’s horse racing optimization (THRO) algorithm.

**Fig. 20:**
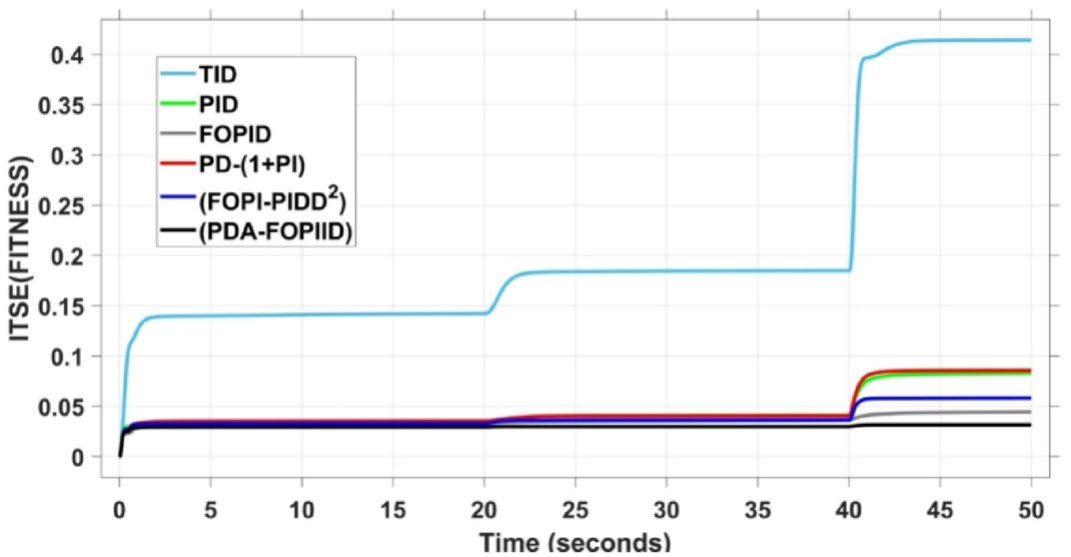
ITSE Vs time with case II.

In the context of the combined LFC-AVR system, the evaluation concentrates on reducing key dynamic performance indices, including overshoot, undershoot, and settling time of frequency deviations in both areas. As noted in Fig. 17, the proposed regulator achieves significantly outperform results, with overshoot values of $\:10.9\:\times\:\:{10}^{-3}$ and $\:4.5\:\times\:\:{10}^{-3}$, undershoot values of $\:-23.1\:\times\:\:{10}^{-3}$ and $\:-21\:\times\:\:{10}^{-3},$ and settling times of $\:2.8$ and $\:2.5\:seconds$ for ΔF_a_ and ΔF_b_, respectively. These results clearly indicate the controller’s ability to maintain frequency stability under successive disturbances. The variation response of tie-line power, illustrated in Fig. 18, further demonstrates the efficiency of the proposed PDA-FOPIID controller in mitigating fluctuations. The controller facilitates a rapid return to steady-state conditions during both stages of the disturbance, with a notably short settling time of $\:3\:seconds$. This performance emphasizes the controller’s robustness and responsiveness during system instability. Moreover, as depicted in Fig. 19, the impact of the multi-step disturbances on terminal voltage deviations is not significantly greater than that observed in the initial disturbance scenario. The PDA-FOPIID controller again exhibits the most favorable performance among the compared designs. For ΔVₐ and ΔV_b_, the controller maintains maximum overshoot values of $\:1.066$ and $\:1.065$, settling times of $\:1.4$ and $\:2.0\:seconds$, and rise times of 0.325 s in both cases. These findings confirm the controller’s ability to sustain voltage stability, even in more complex and demanding disturbance scenarios. Further insights are drawn from Table 4, which documents the system’s response metrics and supports the conclusion that the PDA-FOPIID controller significantly outperforms alternative designs. It demonstrates enhanced attenuation of frequency deviation overshoots and undershoots, as well as enhanced damping of tie-line power deviations. Additionally, Fig. 20 shows that during the disturbance conditions of Scenario 2, the proposed controller achieves the best fitness value of $\:0.0314$. A detailed comparison of robustness and fitness values across all controllers is provided in Table 5.

In conclusion, the intelligent PDA-FOPIID regulator, fine-tuned using the THRO algorithm, demonstrates remarkable improvements in system performance when compared to its MSO-tuned counterparts. Specifically, it delivers performance enhancements of $\:62.5\%$ over TID, $\:60.5\%$ over PID, $\:27\%$ over FOPID, $\:61\%$ over PD-(1 + PI), and $\:54\%$ over FOPI-PIDD². These findings validate the effectiveness and reliability of the proposed PDA-FOPIID regulator for maintaining the stability of both frequency and voltage in two-area power systems under complex and sequential load disturbances.

**Table 4:** The dynamic characteristics of the compared controllers with case II.

Controllers		TID	PID	FOPID	PD-(1 + PI)	FOPI-PIDD^2^	PDA-FOPIID (Proposed)
Δfa	**MOS**	0.0674	0.016	0.035	0.0191	0.0233	0.0109
	**MUS**	−0.138	−0.086	−0.0337	−0.0949	−0.0897	−0.0231
	**ST**	7	8	16	14	12	2.8
ΔFb	**MOS**	0.0449	0.01	0.0077	0.011	0.0123	0.0045
	**MUS**	−0.224	−0.0825	−0.0369	−0.184	−0.154	−0.021
	**ST**	6	6.5	10	5.5	11	2.5
ΔPtie	**MOS** $\:\times\:{10}^{-3}$	36.3	20.1	4.66	20.8	16.4	12.9
	**MUS** $\:\:\times\:{10}^{-3}$	−8.9	−1.2	0	−2.35	−1.1	0
	**ST**	17	15	16	29	31	3
ΔVa	**MOS**	1.143	1.227	1.106	1.096	1.07	1.066
	**RT**	0.49	0.162	0.27	0.7	0.311	0.325
	**ST**	3	3.5	3.7	2.3	3.5	1.4
ΔVb	**MOS**	1.44	1.255	1.171	1.077	1.086	1.065
	**RT**	0.5	0.15	0.24	0.75	0.27	0.325
	**ST**	3	3.5	2.7	3.2	3	2

**Table 5:** Fitness values for compared controllers with scenario II.

Controllers	Fitness				
	ΔFa	ΔFb	ΔVa	ΔVb	Total
TID	0.05396	0.2331	0.06268	0.06441	0.4142
PID	0.006403	0.0435	0.01669	0.01643	0.08302
FOPID	0.001954	0.007929	0.01652	0.0181	0.0445
PD-(1 + PI)	0.006424	0.04774	0.01579	0.01575	0.0857
FOPI-PIDD^2^	0.003986	0.0232	0.01568	0.01545	0.05831
PDA-FOPIID (Proposed)	0.000438	0.001964	0.01479	0.01423	0.03141

C. **Scenario III: A load disturbance is randomly subjected to Area A**.

**Fig. 21:**
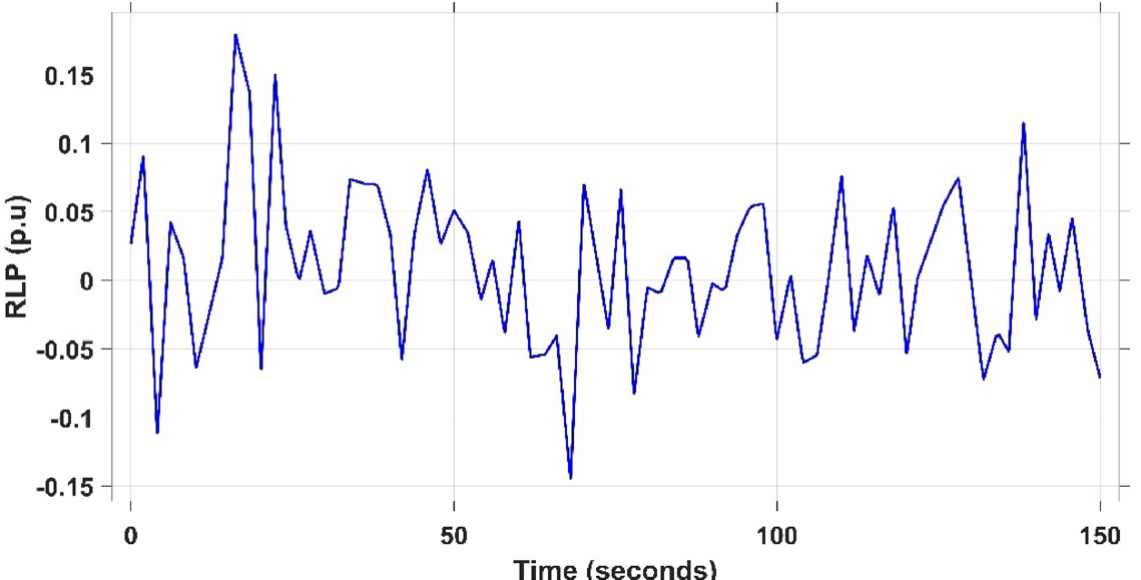
The load/generation patterns for Scenario 3.

**Fig. 22:**
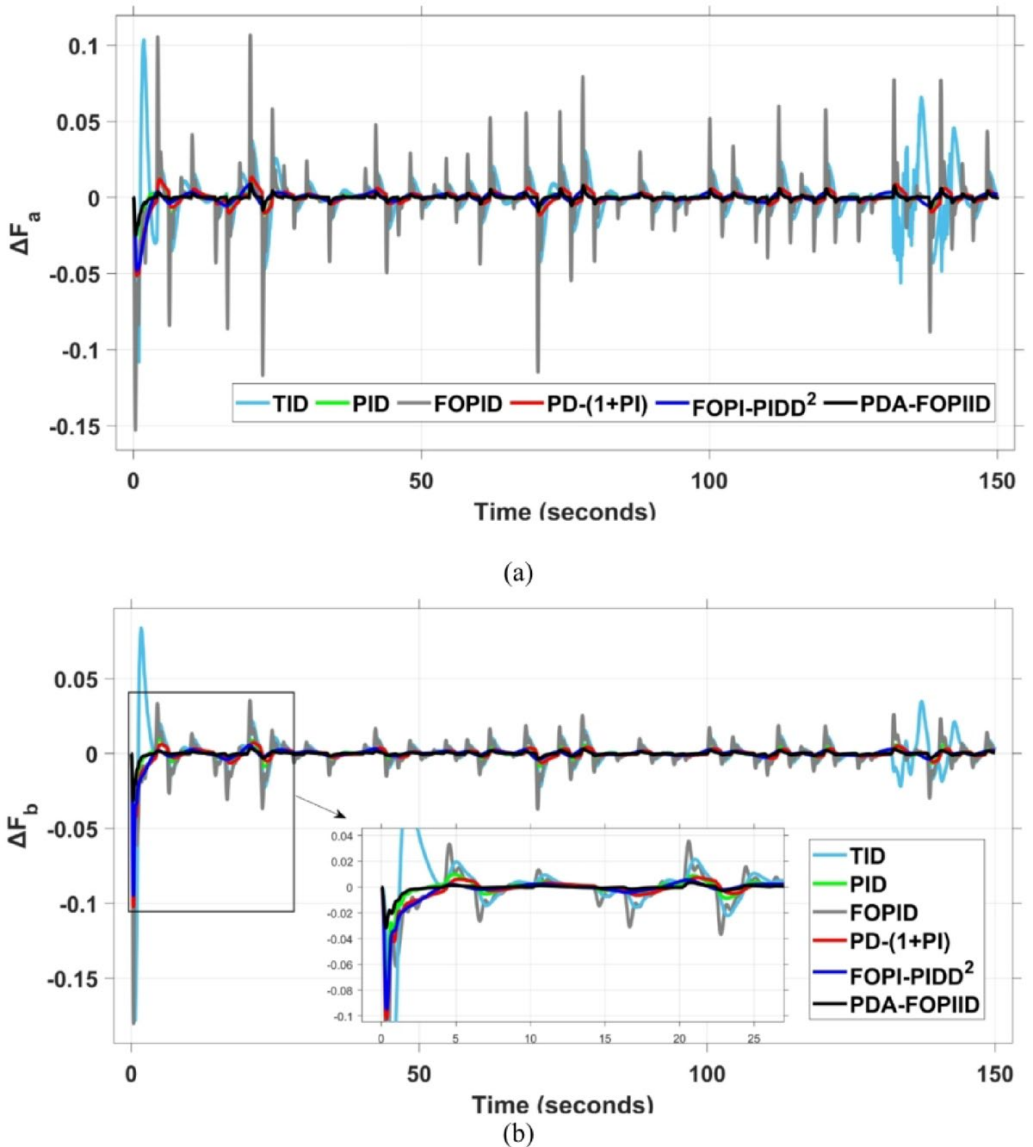
Frequency curves based on case III characteristics- (**a**) ΔF_a_, (**b**) ΔF_b_.

**Fig. 23:**
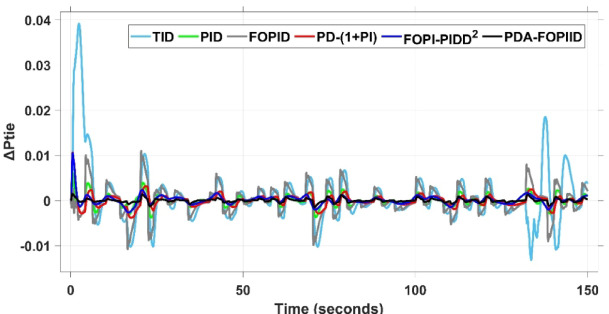
ΔPtie curves based on case III.

This case is designed to assess the behavior of the presented PDA-FOPIID controller, coordinated with the electric vehicle (EV) system, in enhancing the Load Frequency Control (LFC) and Automatic Voltage Regulation (AVR) loops of a two-area interconnected power system. To emulate a realistic operational condition, a load variation is randomly subjected to Area A of the system, as illustrated in the load pattern shown in Fig. 21, allowing for the evaluation of the regulator’s dynamic adaptability. This scenario closely reflects real-world situations where power demand fluctuates continuously throughout the day based on the consumption patterns in each area of the power grid. The simulation outcomes for frequency deviations under this variable load condition are depicted in Fig. 22, where the behavior of the presented PDA-FOPIID regulator is tested with several advanced controllers, including TID, PID, FOPID, PD-(1 + PI), and FOPI-PIDD². As shown in Fig. 23, the response of tie-line power (ΔPtie) clearly demonstrates that the presented regulator offers the fastest return to steady-state conditions. This rapid stabilization translates to a shorter total system settling time and thus enhances overall system resilience.

**Fig. 24:**
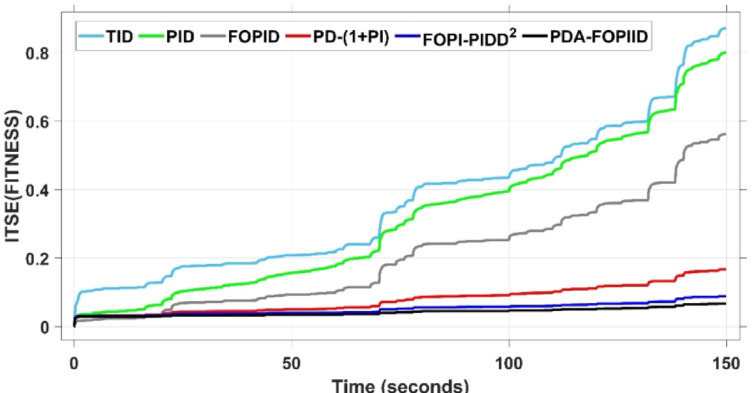
ITSE Vs time with case III.

**Fig. 25:**
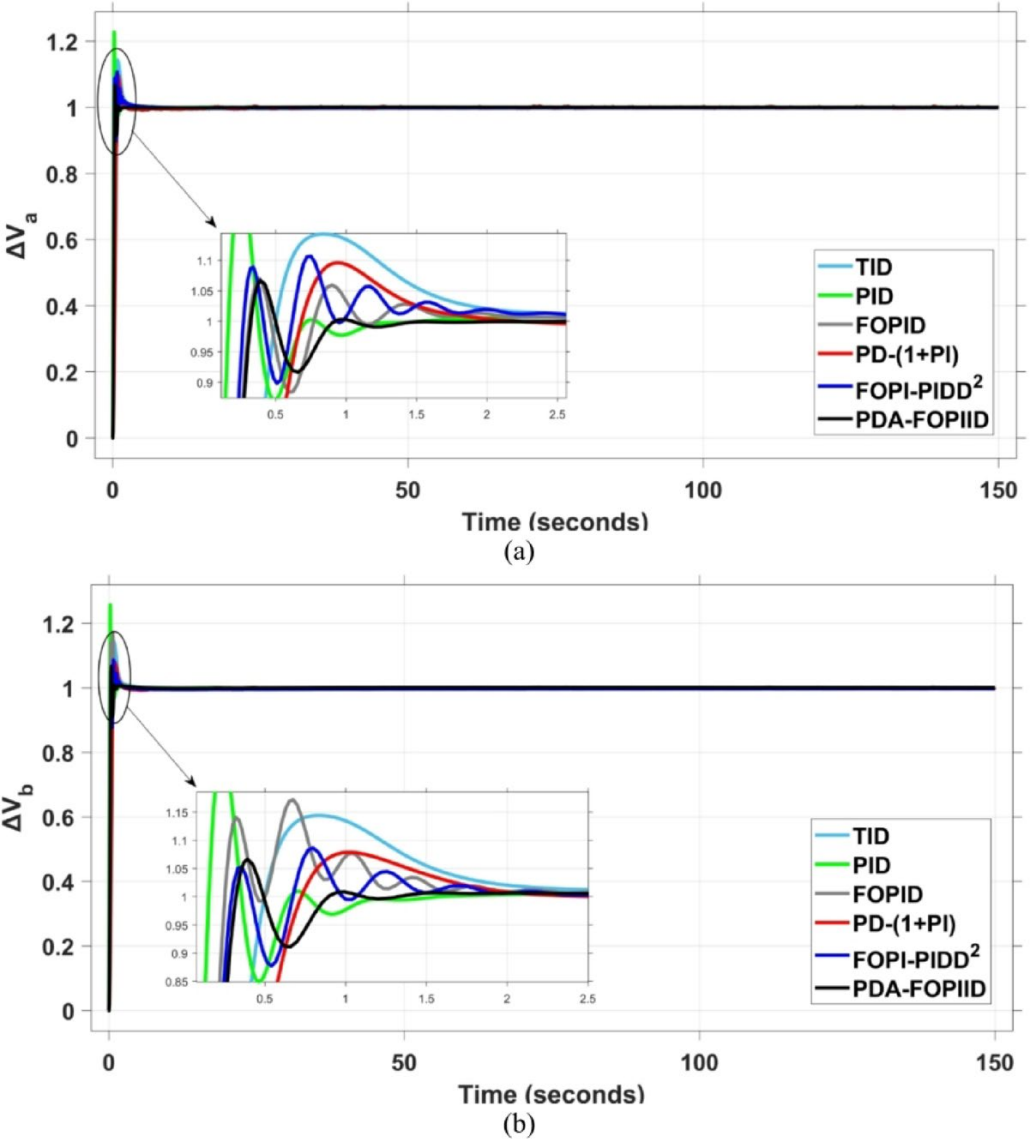
Voltage curves based on case III characteristics - (**a**) ΔV_a_, (**b**) ΔV_b_.

The results indicate that the proposed coordination strategy significantly outperforms the other individual and hybrid controllers in mitigating severe frequency and power fluctuations caused by random load changes. It effectively restrains oscillations while ensuring quick recovery. Specifically, the controller limits frequency deviations within the range of $\:-0.024$ Hz to $\:+0.008$ Hz with respect to Area A, and $\:-0.03$ Hz to $\:+0.002$ Hz with respect to Area B, while maintaining tie-line power fluctuations within $\:\pm\:0.0014\:p.u$. These minimal deviations contribute to achieving the best fitness value of $\:0.0673$, as shown in Fig. 24, outperforming all other tested controllers. The detailed fitness values of all compared methods are presented in Table 6. Regarding terminal voltage deviations, Fig. 25 shows that voltage was not significantly affected during the disturbance period and remained within a negligible range throughout the simulation. However, at the initial stage of the simulation, the presented regulator achieved the lowest maximum overshoot (MOS) values of $\:1.067$ and $\:1.066$, and settling times of $\:2.7\:s$ and $\:5.0\:s$ for ΔV_a_ and ΔV_b_, respectively. The outcomes highlight the controller’s robustness in maintaining stable voltage levels, even during transients.

In conclusion, the PDA-FOPIID controller, when integrated with EV participation and optimized through the THRO algorithm, demonstrates superior performance compared to other advanced control strategies. It achieves the lowest overshoot and shortest settling times for frequency, tie-line power, and voltage deviations under random load disturbances and uncertainty. A comprehensive summary of these findings is provided in Table 7, further reinforcing the controller’s robustness and suitability for practical implementation in modern multi-area power systems.

**Table 6:** Fitness values for compared controller in case III.

Controllers	Fitness				
	ΔFa	ΔFb	ΔVa	ΔVb	Total
TID	0.363334	0.3591	0.0812	0.0674	0.871034
PID	0.346267	0.3193	0.0726	0.0631	0.801267
FOPID	0.24446	0.2235	0.0526	0.0417	0.56226
PD-(1 + PI)	0.069493	0.0635	0.0193	0.0151	0.167393
FOPI-PIDD^2^	0.035953	0.0335	0.0102	0.0087	0.088353
PDA-FOPIID (Proposed)	0.0279109	0.0226	0.0093	0.0075	0.0673109

**Table 7:** The metric values of the compared controllers with case III.

Controllers		TID	PID	FOPID	PD-(1 + PI)	FOPI-PIDD^2^	PDA-FOPIID (Proposed)
Δfa	**MOS**	0.103	0.017	0.106	0.013	0.008	0.008
	**MUS**	−0.108	−0.049	−0.152	−0.051	−0.047	−0.024
ΔFb	**MOS**	0.0837	0.0094	0.0327	0.006	0.003	0.002
	**MUS**	−0.178	−0.076	−0.18	−0.0101	−0.094	−0.03
ΔPtie	**MOS**	0.039	0.006	0.0109	0.0022	0.0105	0.0014
	**MUS**	−0.013	−0.0036	−0.0107	−0.003	−0.002	−0.0014
ΔVa	**MOS**	1.141	1.223	1.07	1.095	1.1068	1.067
	**RT**	0.0515	0.162	0.29	0.0684	0.265	0.325
	**ST**	12	20	19	17	15	2.7
ΔVb	**MOS**	1.44	1.25	1.171	1.07	1.08	1.066
	**RT**	0.48	0.15	0.25	0.73	0.29	0.32
	**ST**	11	20	12	21	19	5

D. **Scenario IV: Applying a variation of RESs**.

**Fig. 26:**
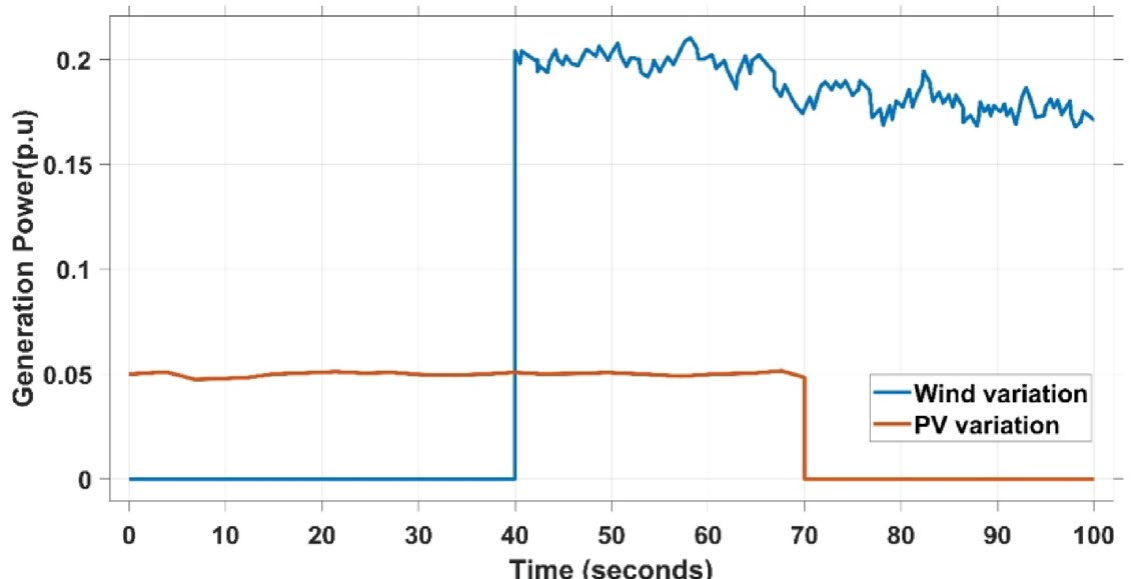
The loading/generation profiles case IV.

This scenario presents the effectiveness of the suggested PDA-FOPIID collaboration strategy along with the contribution of electric vehicles (EVs) is validated by introducing the effects of renewable energy source (RES) variation and intermittency into the considered dual-area microgrid. Accordingly, wind and photovoltaic (PV) plants are integrated into the two areas, as illustrated in Fig. 1. The fluctuations in PV and wind are shown in Fig. 26, while the regulator’ performance metrics for this case are reported in Table 8.

**Fig. 27:**
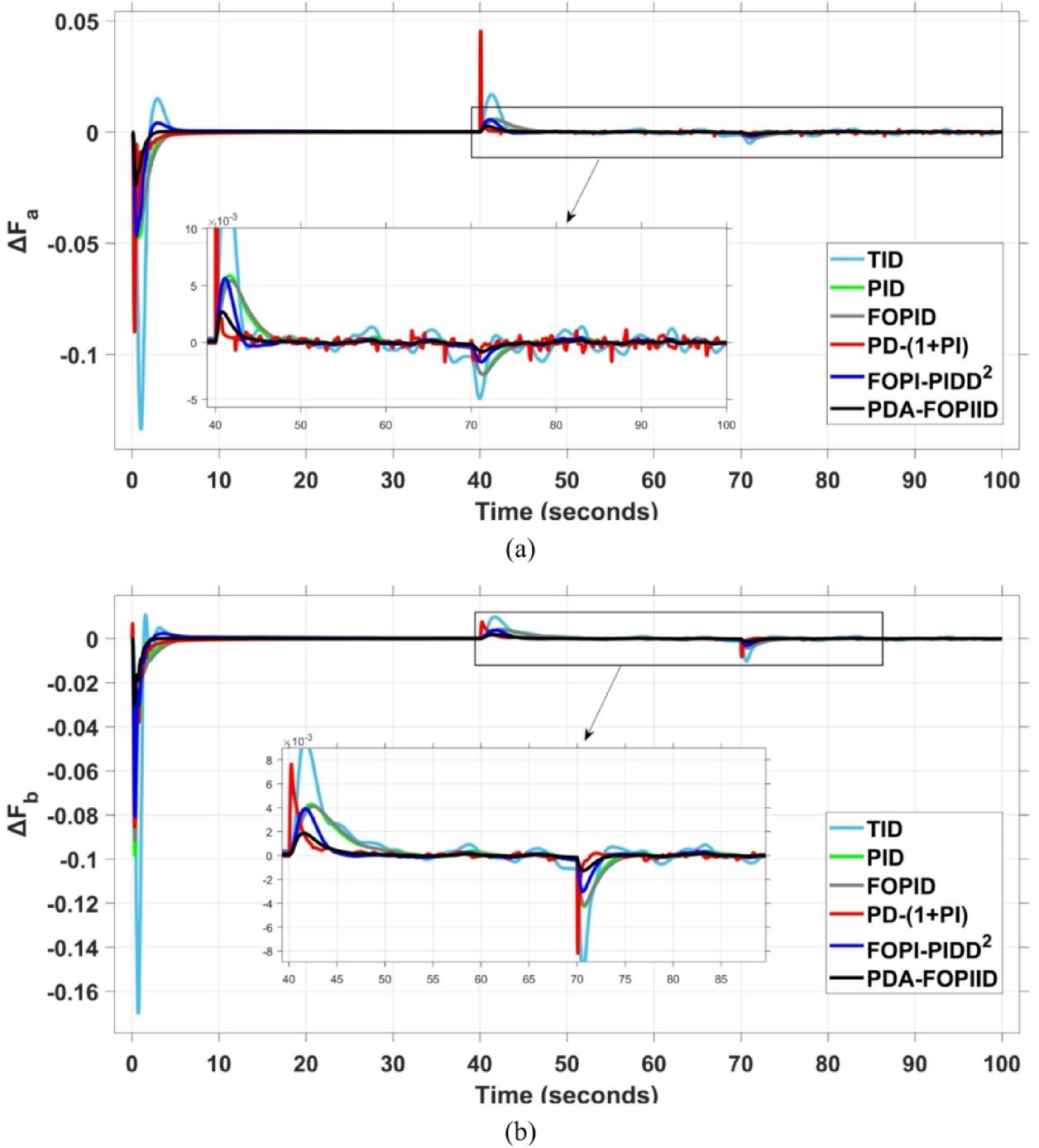
Frequency curves based on case IV characteristics- (**a**) ΔF_a_, (**b**) ΔF_b_.

**Fig. 28:**
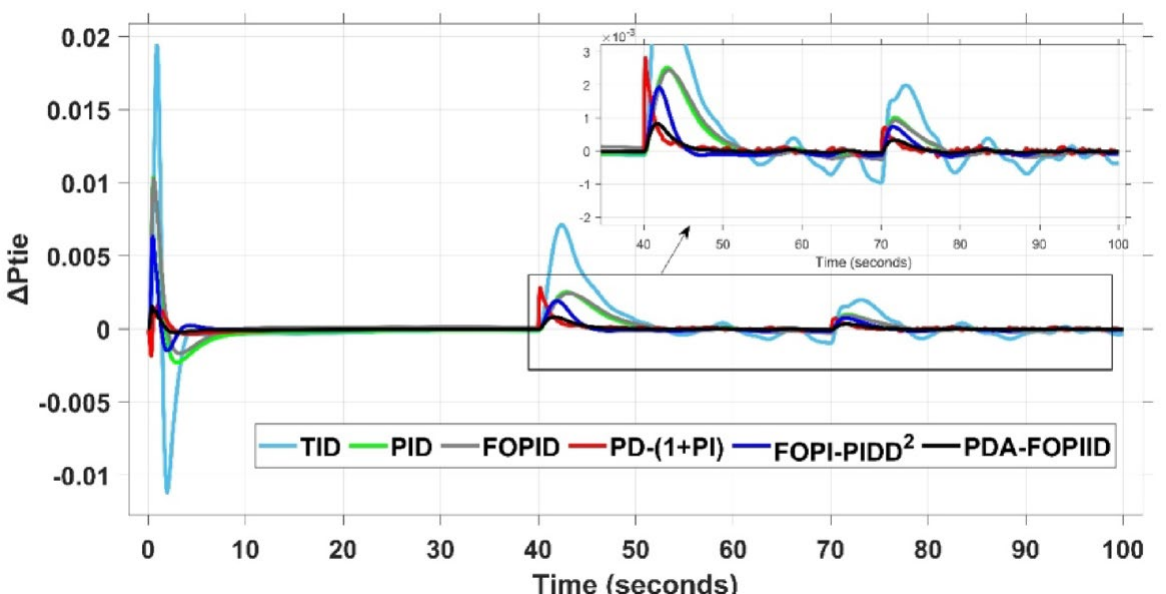
ΔPtie curves based on case IV.

**Fig. 29:**
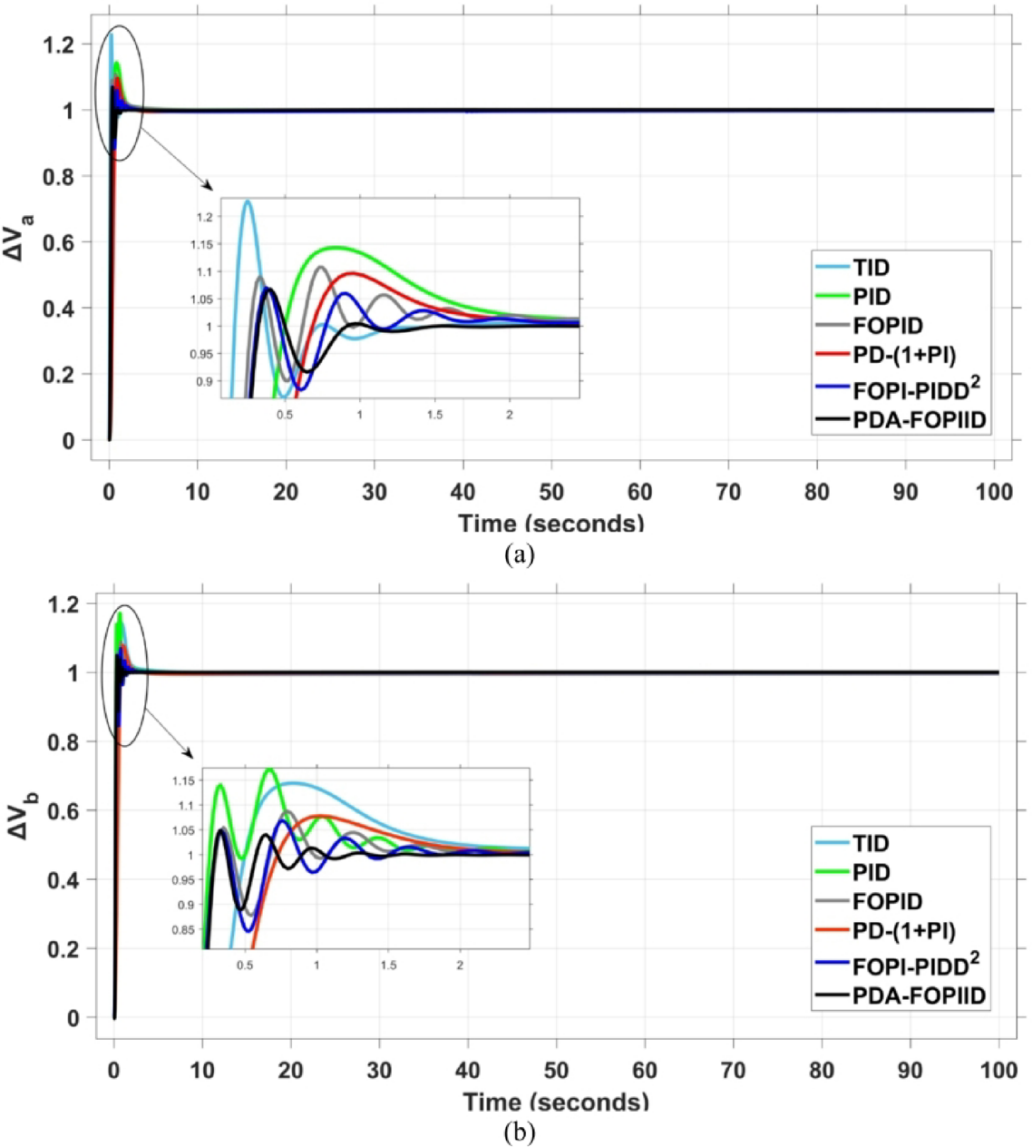
Voltage curves based on case IV characteristics- (**a**) ΔV_a_, (**b**) ΔV_b_.

The PV output is connected at the starting time ($\:t\:=\:0\:s$) and cancelled at $\:t\:=\:70\:s$, whereas the wind output is introduced at $\:t\:=\:40\:s$. Figures 27 and 28, and 29 depict the dynamic responses of the two areas under these disturbances. As shown in Fig. 27, frequency deviations temporarily increase at the beginning of the simulation due to the sudden input from PV generation and the activation of the AVR system. This is especially notable with the conventional TID controller, which shows maximum undershoot (MUS) values of approximately $\:-0.133$ Hz and $\:-0.169$ Hz in areas A and B, respectively, at $\:t\:=\:0\:s$. Upon the wind farm’s integration at $\:t\:=\:40$ s, the maximum overshoot (MOS) reaches around $\:16.8\times\:{10}^{-3}$ Hz and $\:9.7\times\:{10}^{-3}$ Hz in areas A and B, respectively. These deviations then significantly drop to $\:-4.9\times\:{10}^{-3}$ Hz and $\:-10.1\times\:{10}^{-3}$ Hz at $\:t\:=\:70$ s following the PV disconnection.

**Fig. 30:**
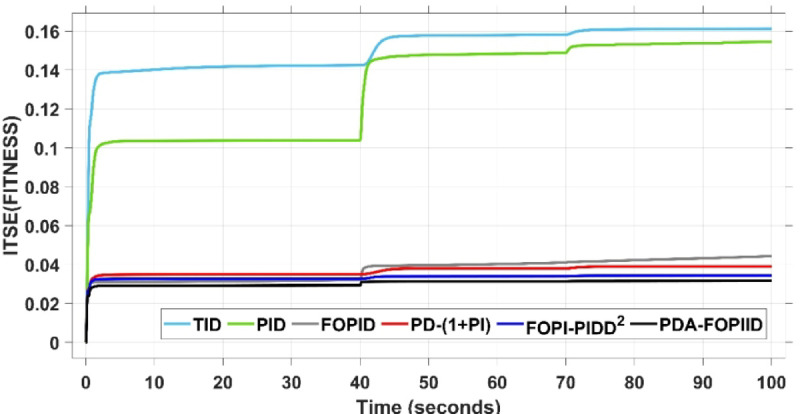
ITSE Vs time with scenario IV.

In contrast, the proposed PDA-FOPIID controller demonstrates superior efficiency and faster frequency restoration. At $\:t\:=\:0\:s$, MUS values are considerably lower around $\:-0.03$ Hz and $\:-0.02$ Hz for areas A and B, respectively. During wind integration at $\:t\:=\:40\:s$, the MOS values are also reduced to $\:2.6\times\:{10}^{-3}$ Hz and $\:1.8\times\:{10}^{-3}$ Hz, then decline further to $\:-0.8\times\:{10}^{-3}$ Hz and $\:-1.1\times\:{10}^{-3}$ Hz at $\:t\:=\:70\:s$ after the PV system is disconnected. Regarding tie-line power deviation (ΔPtie), Fig. 28 shows that the proposed controller achieves the most favorable response, with a MOS of approximately $\:1.4\:p.u$. and zero MUS, reaching steady state within the shortest settling time (ST) of $\:7\:seconds$ compared to other controllers namely TID, PID, FOPID, PD-(1 + PI), and FOPI-PIDD². Finally, for terminal voltage deviation (ΔV_a_ and ΔV_b_), Fig. 29 confirms the superior performance of the proposed controller once again. It achieves the lowest MOS values of $\:1.066$ and $\:1.065$, with settling times of $\:1.4\:s$ and $\:2.0\:s$, and rise times (RT) of $\:0.325\:s$ for both areas. Figure 30 illustrates the improved effectiveness of the presented regulator, which achieved the optimal fitness value of $\:0.03162$, outperforming all other controllers listed in Table 9.

It can be concluded that under scenarios involving RES variations, the proposed PDA-FOPIID controller tuned by THRO consistently outperforms the other compared controllers with respect to maximum overshoot (MOS), maximum undershoot (MUS), rise time (RT), and settling time (ST). Specifically, it delivers performance gains include 55% vs. TID, 41% vs. PID, 50% vs. FOPID, 65% vs. PD-(1 + PI), and 50% vs. FOPI-PIDD².

**Table 8:** The dynamic characteristics of the compared controllers with scenario IV.

Controllers		TID	PID	FOPID	PD-(1 + PI)	FOPI-PIDD^2^	PDA-FOPIID (Proposed)
Δf_a_	**MOS**	0.0168	0.0058	0.0054	0.044	0.0056	0.0026
	**MUS**	−0.133	−0.048	−0.044	−0.089	−0.044	−0.023
	**ST**	8	6	6	14	10	3
ΔF_b_	**MOS**	0.0105	0.0042	0.004	0.0075	0.0038	0.0018
	**MUS**	−0.0169	−0.098	−0.091	−0.085	−0.081	−0.028
	**ST**	54	7	7	15	15	2.5
ΔPtie	**MOS** $\:\times\:{10}^{-3}$	19.0	10.4	10.2	2.4	6.0	1.4
	**MUS** $\:\:\times\:{10}^{-3}$	−11.0	−2.2	−1.6	−1.7	−1.4	0
	**ST**	25	53	56	25	10	7
ΔV_a_	**MOS**	1.143	1.227	1.106	1.096	1.07	1.066
	**MUS**	0.49	0.162	0.27	0.7	0.311	0.325
	**ST**	3	3.5	3.7	2.3	3.5	1.4
ΔV_b_	**MOS**	1.44	1.255	1.171	1.077	1.086	1.065
	**RT**	0.5	0.15	0.24	0.75	0.27	0.325
	**ST**	3	3.5	2.7	3.2	3	2

**Table 9:** Fitness values for compared controller with scenario IV.

Controllers	Fitness				
	ΔFa	ΔFb	ΔVa	ΔVb	Total
TID	0.062	0.047	0.03	0.0223	0.16127
PID	0.045	0.06	0.027	0.0225	0.15455
FOPID	0.017	0.0135	0.00577	0.008	0.04427
PD-(1 + PI)	0.015	0.0116	0.00536	0.007	0.03896
FOPI-PIDD^2^	0.013	0.0102	0.00476	0.0063	0.03426
PDA-FOPIID (Proposed)	0.012	0.00412	0.0095	0.006	0.03162

E. **Scenario V: Implementing a high level of RES integration**.

**Fig. 31:**
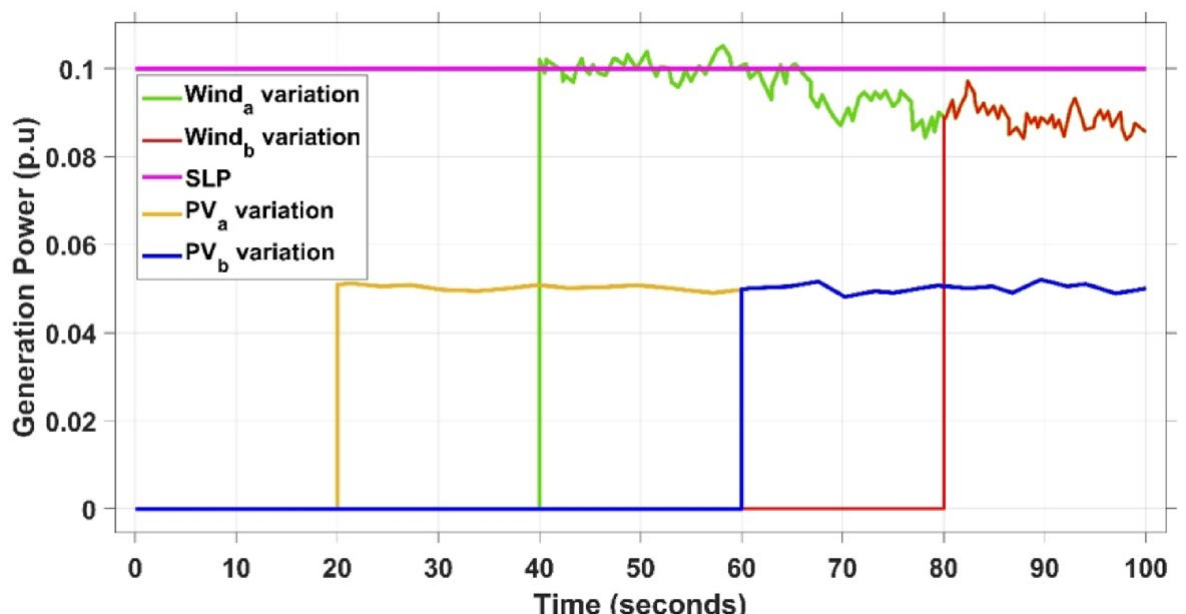
The loading/generation pattern case V.

This scenario evaluates the behavior of the newly presented PDA-FOPIID controller, optimized using the THRO algorithm, and its coordination with electric vehicles (EVs) in both the load frequency control (LFC) and automatic voltage regulation (AVR) loops. A comprehensive test case is established to assess the strength of the multi-area microgrid (MG) system amid the uncertainties introduced by high renewable energy source (RESs) penetration. The simulation begins with a $\:1\%$ step disturbance to the load (SLD), followed by the sequential integration of RES units: the PV plant in Area A at $\:t\:=\:20\:s$, the PV plant in Area B at $\:t\:=\:60\:s$, the wind farm in Area A at $\:t\:=\:40\:s$, and the wind farm in Area B at $\:t\:=\:80\:s$. The fluctuations in wind, PV, and load power are illustrated in Fig. 31. The performance outcomes, demonstrating the robustness of the presented regulator, are summarized in Table 10.

**Fig. 32:**
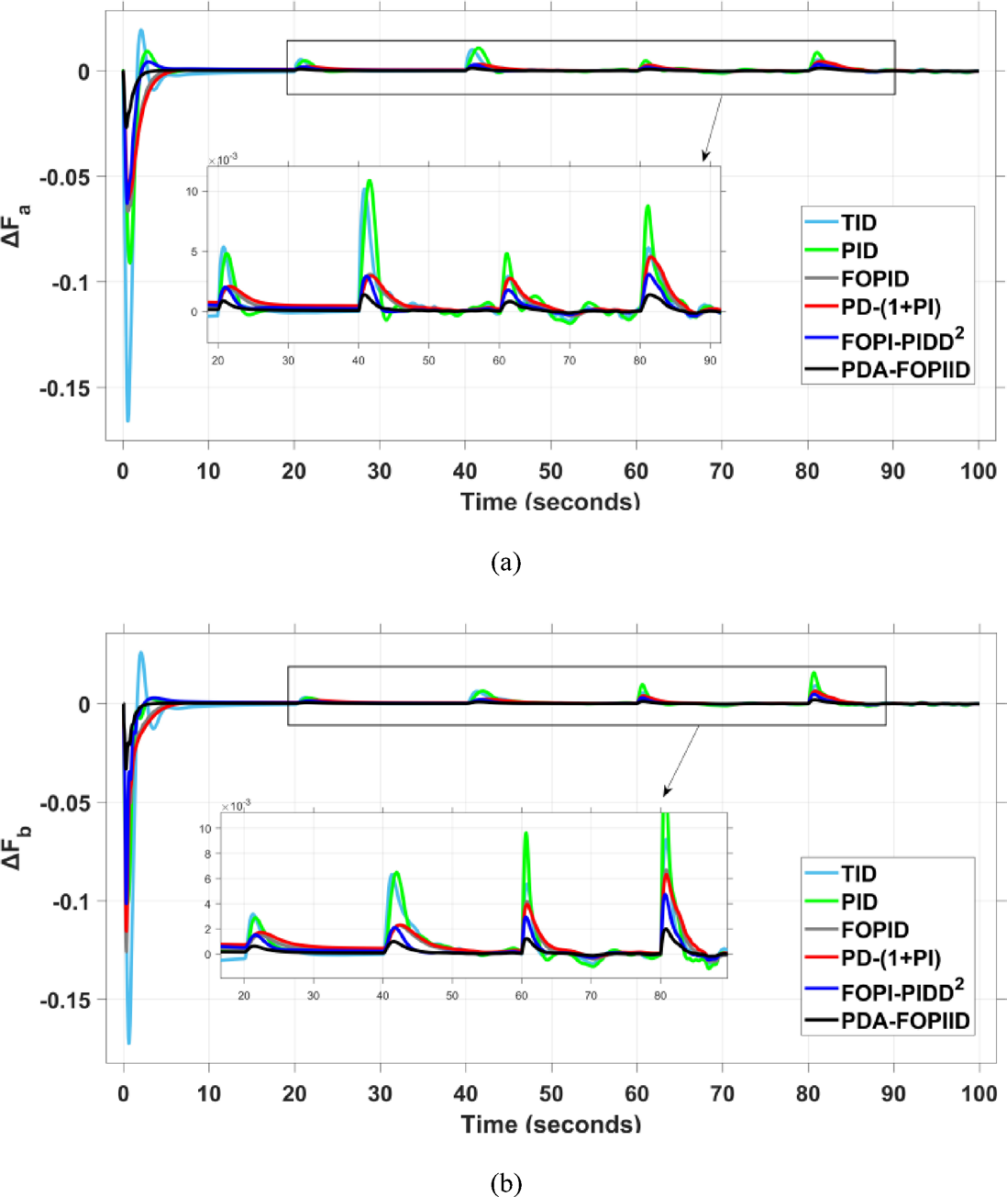
Frequency curves based on case V characteristics- (**a**) ΔF_a_, (**b**) ΔF_b_.

As illustrated in Fig. 32, frequency deviations temporarily increase at the start of the simulation due to the sudden impact of the step load disturbance (SLD) and the activation of the AVR system. Despite these disturbances, the proposed PDA-FOPIID controller exhibits superior efficiency and faster frequency restoration. At $\:t\:=\:0\:s$, the maximum undershoot (MUS) values are significantly reduced to approximately $\:-0.02$6 Hz and $\:-0.033$ Hz corresponding to Areas A and B. During the PV integration in Area A at $\:t\:=\:20\:s$, the maximum overshoot (MOS) values are as low as $\:0.8\times\:{10}^{-3}$ Hz and $\:0.5\times\:{10}^{-3}$ Hz. Similarly, during wind integration in Area A at $\:t\:=\:40\:s$, the MOS values remain low at $\:1.3\times\:{10}^{-3}$ Hz and $\:0.9\times\:{10}^{-3}$ Hz. A slight rise is observed at $\:t\:=\:60\:s$ following PV integration from Area B, with values of $\:0.8\times\:{10}^{-3}$ Hz and $\:1.2\times\:{10}^{-3}$ Hz. The final disturbance occurs at $\:t\:=\:80\:s$ when the wind farm in Area B is connected, causing a modest increase in frequency deviations to $\:1.35\times\:{10}^{-3}$ Hz and $\:1.9\times\:{10}^{-3}$ Hz.

**Fig. 33:**
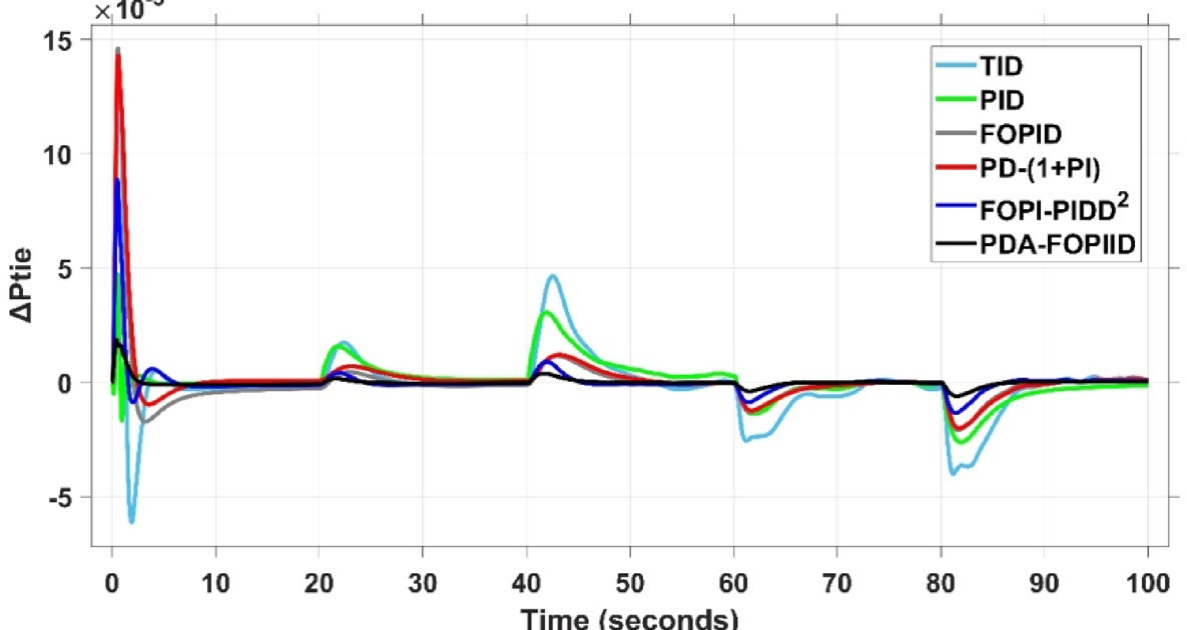
ΔPtie curves based on case scenario V.

**Fig. 34:**
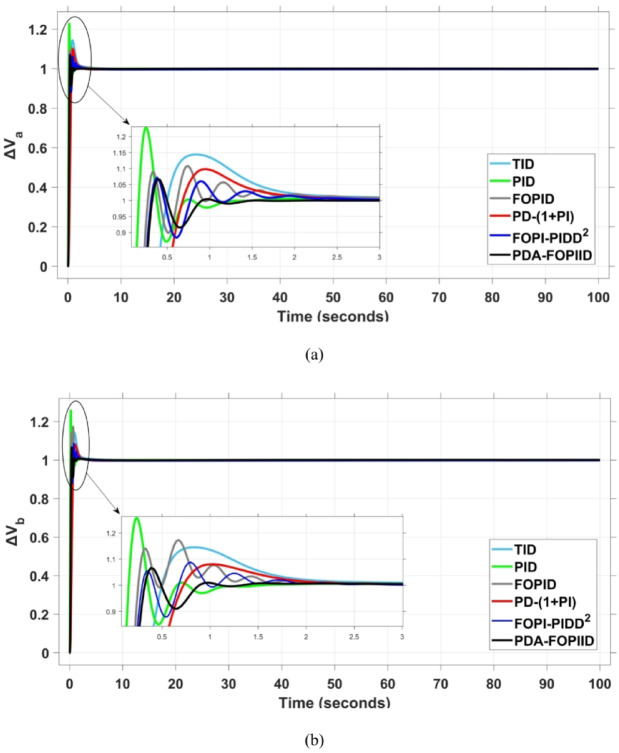
Voltage curves based on case V characteristics- (a) ΔV_a_, (b) ΔV_b_.

**Fig. 35:**
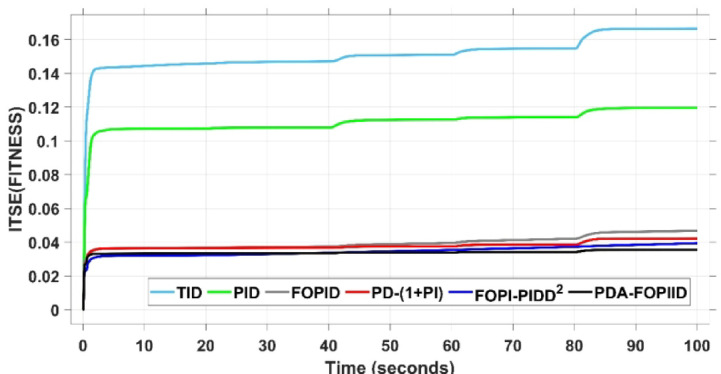
ITSE Vs time with scenario V.

With respect to tie-line power deviation (ΔPtie), Fig. 33 demonstrates that the proposed controller achieves the most stable and responsive performance, registering a MOS of approximately $\:1.8\times\:{10}^{-3}p.u.$ and a MUS of $\:0.5\times\:{10}^{-3}p.u.$, along with the fastest return to steady-state conditions when compared to other controllers, including TID, PID, FOPID, PD-(1 + PI), and FOPI-PIDD². For terminal voltage deviations (ΔV_a_ and ΔV_b_), Fig. 34 confirms the superior performance of the proposed controller once again, as its response remains consistent with that observed in the previous scenario. It achieves the lowest MOS values of $\:1.066$ and $\:1.065$, with settling times of $\:1.4\:s$ and $\:2.0\:s$, and rise times (RT) of $\:0.325\:s$ for both areas. Figure 35 further highlights the exceptional behavior of the presented regulator, which achieved the lowest fitness value of $\:0.0354$, outperforming all other controllers listed in Table 11.

In conclusion, under scenarios involving high penetration of RES, the presented PDA-FOPIID regulator tuned by THRO consistently demonstrates superior performance across key metrics maximum overshoot (MOS), maximum undershoot (MUS), rise time (RT), and settling time (ST). Specifically, compared to TID, PID, FOPID, PD-(1 + PI), and FOPI-PIDD², the controller achieves respective improvements of 65%, 31%, 45%, 57%, and 46%.

**Table 10:** The metric values of the compared controllers with scenario V.

Controllers		TID	PID	FOPID	PD-(1 + PI)	FOPI-PIDD^2^	PDA-FOPIID (Proposed)
Δf_a_	**MOS**	0.019	0.0108	0.003	0.0071	0.0025	0.0013
	**MUS**	−0.165	−0.091	−0.066	−0.060	−0.062	−0.026
	**ST**	15	7	5	6	8	3.5
ΔF_b_	**MOS**	0.025	0.015	0.006	0.006	0.0045	0.0015
	**MUS**	−0.172	−0.101	−0.125	−0.115	−0.101	−0.033
	**ST**	-	6	7	7	10	3.5
ΔPtie	**MOS** $\:\times\:{10}^{-3}$	0.0130	0.003	0.0145	0.0141	0.0086	0.0046
	**MUS** $\:\:\times\:{10}^{-3}$	−6.1	−1.6	−2.0	−1.2	−1.0	−0.3
ΔV_a_	**MOS**	1.143	1.227	1.106	1.096	1.07	1.066
	**MUS**	0.49	0.162	0.27	0.7	0.311	0.325
	**ST**	3	3.5	3.7	2.3	3.5	1.4
ΔV_b_	**MOS**	1.44	1.255	1.171	1.077	1.086	1.065
	**RT**	0.5	0.15	0.24	0.75	0.27	0.325
	**ST**	3	3.5	2.7	3.2	3	2

**Table 11:** Fitness values for compared controller with scenario V.

Controllers	Fitness				
	ΔF_a_	ΔF_b_	ΔV_a_	ΔV_b_	Total
TID	0.0626	0.0487	0.0318	0.0235	0.1666
PID	0.0426	0.0319	0.0267	0.0184	0.1196
FOPID	0.0182	0.0124	0.0095	0.0067	0.0468
PD-(1 + PI)	0.0151	0.0112	0.0093	0.0071	0.0421
FOPI-PIDD^2^	0.0143	0.0104	0.0085	0.0062	0.0394
PDA-FOPIID (Proposed)	0.0121	0.0096	0.0078	0.0059	0.0354

**Fig. 36:**
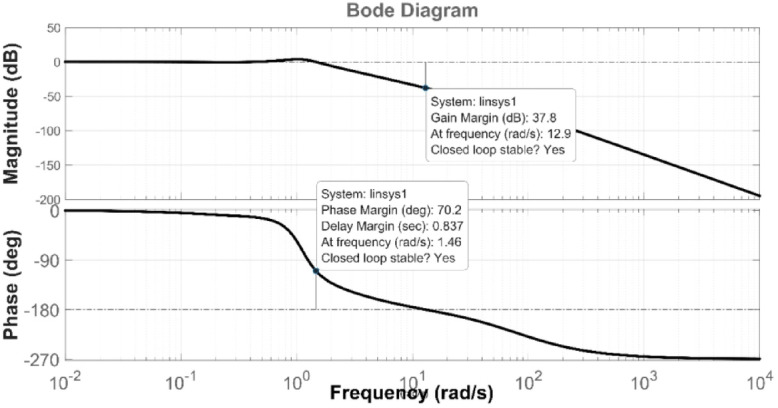
Frequency response (Bode plot) of the proposed PDA-FOPIID controller.

To validate the stability of the system under the proposed PDA-FOPIID control strategy, a frequency response analysis was conducted, and the corresponding Bode plots are presented in Fig. 36. The results confirm that the closed-loop system remains stable, demonstrating a gain margin of 37.8 dB and a phase margin of 70.2°. Additionally, the delay margin of 0.837 s further indicates that the system can tolerate considerable disturbances without losing stability. These findings clearly highlight the effectiveness of the proposed controller in enhancing the robustness and stability of the interconnected power system.

## Conclusions

Integrating the (AVR) loop with the (LFC) loop through coupling coefficients enables coordinated control of voltage and frequency, which is especially important in hybrid microgrids incorporating electric vehicle (EV) models. In this work, both control loops are simultaneously regulated using a novel PDA-FOPIID controller, functioning as a secondary control mechanism. This controller is fine-tuned using the Tianji’s Horse Racing Optimization (THRO) algorithm, ensuring robust and dynamic performance across varying system conditions. Its effectiveness was validated on a two-area hybrid power system. To further enhance its performance, the presented controller was tuned by several metaheuristic algorithms, including GTO, PSO, WHO, and GBO. Among these, the THRO algorithm yielded the most effective results, offering faster convergence and improved response characteristics. The performance of the THRO-optimized PDA-FOPIID controller was compared with several other THRO-based regulators, like TID, FOPI-PIDD², PD-(1 + PI), FOPID, and the conventional PID.

The regulator’s capabilities were tested under diverse scenarios, such as a 1% step disturbance to the load in Area A, multi-step load changes across both areas, random load fluctuations, the implementation of renewable energy sources (RESs), and high RES penetration conditions. Across all cases, the PDA-FOPIID controller consistently outperformed its counterparts in controlling system frequency, tie-line power flow, and voltage variations. The results demonstrate its superior ability to enhance frequency stability and overall dynamic performance in dual-area hybrid microgrids. The proposed PDA-FOPIID controller, optimized using the Tianji’s Horse Racing Optimization (THRO) algorithm, demonstrated notable performance improvements across critical control parameters. In terms of frequency variation (ΔF), tie-line power variation (ΔPtie), and voltage deviation (ΔV), the controller consistently outperformed other THRO-tuned counterparts. Specifically, it achieved enhancements of 38% over FOPID, 62% over TID, 60% over PD-(1 + PI), 45% over conventional PID, and 52% over FOPI-PIDD². These results highlight the controller’s effectiveness in mitigating system disturbances and maintaining stability under dynamic operating conditions. Additionally, sensitivity analysis confirmed the controller’s stability and responsiveness in a wide range of different cases.

Despite its advantages, the presented controller presents several impacts. These factors contribute to a higher processing burden than that of simpler controllers, a high degree of parameter tuning complexity due to its dimensionality, and occasional underperformance when compared to intelligent control methods such as fuzzy logic systems in specific scenarios. To address these limitations, Various strategies for mitigation can be adopted. These involve leveraging higher processors to improve processing efficiency, employing advanced optimization techniques for precise factor tuning, and incorporating a combined tuning approach. In particular, fuzzy logic can be integrated to improve the optimization process of the PDA-FOPIID controller. Fuzzy inference systems can dynamically reshape the search boundaries or refine initial guess of parameters, which are then tuned by metaheuristic approaches such as THRO. This hybrid approach aims to accelerate convergence, reduce processing load, and increase robustness in complicated working conditions. Additionally, some limitations are embedded in the controller’s design. It introduces a not easy hardware, examined under diverse coupling and synchronization parameter settings, nor does it account for the repercussions of delayed communication.

Ongoing and future studies should focus on overcoming these constraints to enhance the practical suitability of the regulator. Key areas for further investigation include incorporating communication delay modeling, extending the control strategy to multi-area power systems, and validating performance through real-time simulation platforms such as OPAL-RT or dSPACE. Furthermore, future work will focus on incorporating the stochastic behavior of EVs, including uncertain arrival/departure times and variable state-of-charge levels, to achieve a more realistic modeling framework. Probabilistic approaches such as Monte Carlo simulation can be employed to better evaluate their impact on frequency and voltage regulation. Exploring these directions will offer a more exhaustive assessment of the regulator’s effectiveness and its feasibility for implementation in realistic and Wide-ranging power system platforms.

## Supplementary Information

Below is the link to the electronic supplementary material.

Supplementary Material 1

## Data Availability

The datasets used and/or analysed during the current study available from the corresponding author on reasonable request.

## Nomenclature

ACE: Area control error
AGC: Automatic generation control
THRO: Tianji’s horse racing optimization
PID: Proportional integral derivative
AVR: Automatic voltage regulation
ITSE: Integral time square error
FOPID: Fractional-order PID
PV: Photovoltaic energy
EV: Electric vehicles
LFC: Load frequency control
SOC: State of charge
ALFC: Automatic load frequency control
FOC: Fractional-order calculus
TID: Tilted integral derivative
LQG: Linear–quadratic–gaussian
ANN: Artificial neural network
ICA: Imperialist competitive algorithm
SO: Seeker optimization
GTO: Gorilla troops optimization
GBO: Whale optimization algorithm
GRC: Generation rate constraints
GDB: Governor dead band
GA: Genetic algorithms
$$\:{T}_{tie}$$: Synchronization coefficient
$\:\varDelta\:{F}_{a}$: Frequency variation in area A
$\:\varDelta\:{F}_{b}$: Frequency variation in area B
$\:\varDelta\:Ptie$: Deviation in power exchange between areas
MOS: Maximum overshoot
MUS: Maximum undershoot
PSO: Particle swarm optimization
ST: Settling time
RT: Rising time
PIDD^2^: Proportional–integral–derivative–double-derivative
PDA: Proportional-derivative-accelerated
FOPIID: Fractional order proportional-integral-double integral-derivative
