# Supplementary material for: Enhanced hybrid microgrid stability with electric vehicle integration using PDA-FOPIID control optimized by tianji’s horse racing algorithm
Source: Sci Rep. 2025 Oct 30;15:37933. doi: 10.1038/s41598-025-22993-1 (PMC12575832; doi:10.1038/s41598-025-22993-1)
Supplement: Supplementary file 1 — Supplementary Material 1 [file 41598_2025_22993_MOESM1_ESM.docx]

**Appendixes**

**Appendix. 1.** System coefficients of Areas A and B in the Simulated Grid

| Parameter | Symbol | Value | |
| --- | --- | --- | --- |
|  |  | **Area A** | **Area B** |
| Rated capacities | $P_{rx}$(MW) | 1200 | 1200 |
| speed regulation | $R_{x}$(Hz/MW) | 2.4 | 2.4 |
| Frequency biases | $\beta_{x}$(MW/Hz) | 0.4249 | 0.4249 |
| Hydraulic governor (time constants) | $T_{gh}$(s) | - | 41.6 |
| Transient droop for hydraulic governor (time constants) | $T_{rh}$(s) | - | 0.513 |
| Hydraulic governor (reset times) | $T_{rs}$(s) | - | 5 |
| Hydro turbine (water starting times) | $T_{w}$(s) | - | 1 |
| Time constant of thermal governor | $T_{sg}$(s) | 0.08 | - |
| Thermal turbines (time constants) | $T_{t}$(s) | 0.3 | - |
| Inertia constants | $H_{x}$(p.u.s) | 0.0833 | 0.0833 |
| Damping coefficients | $D_{x}$(p.u./Hz) | 0.00833 | 0.00833 |
| Wind generation (time constants) | $T_{W}$(s) | 1.5 | - |
| Wind generation (gains) | $K_{W}$ | 1 | - |
| PV generation (time constants) | $T_{PV}$(s) | - | 1.3 |
| PV generation (gains) | $K_{PV}$ | - | 1 |
| Synchronizing coefficient | ${2\pi T}_{tie}$ | 0.55 | |
| EV Models | | | |
| Battery’s SOC (minimum limits) | % | 10 | 10 |
| Voltages (nominal value) | $V_{typ}$(V) | 364.8 | 364.8 |
| Batteries capacities | $C_{typ}$(Ah) | 66.2 | 66.2 |
| Series resistance | $R_{s}$(ohms) | 0.074 | 0.074 |
| Transient resistance | $R_{T}$(ohms) | 0.047 | 0.047 |
| Transient capacitance | $C_{T}$(farad) | 703.6 | 703.6 |
| Constant values | $RT/F$ | 0.02612 | 0.02612 |
| Battery’s SOC (maximum limits) | % | 95 | 95 |
| Battery’s energy capacities | $C_{batt}$(kwh) | 24.15 | 24.15 |

**Appendix. 2**. AVR system coefficients for both areas.

| Model | Transfer function | Parameters | Nominal Values | Parameter Description |
| --- | --- | --- | --- | --- |
| Amplifier | $\frac{K_{A}}{1+T_{A}S}$ | $K_{A}$, $T_{A}$ | 10, 0.1 | Gains and time constants |
| Exciter | $\frac{K_{E}}{1+T_{E}S}$ | $K_{E}$, $T_{E}$ | 1, 0.4 |  |
| Generator (Field CKT) | $\frac{K_{F}}{1+T_{F}S}$ | $K_{F}$, $T_{F}$ | 1, 1 |  |
| Sensor | $\frac{K_{S}}{1+T_{S}S}$ | $K_{S}$, $T_{S}$ | 1, 0.01 |  |

**Appendix. 3**. AVR Coupling confidents.

| Area | α1 | α2 | α3 | α4 | βS |
| --- | --- | --- | --- | --- | --- |
| 1 | 0.3 | 0.1 | 0.5 | 1.4 | 1.5 |
| 2 | 0.3 | 0.1 | 0.5 | 1.4 | 1.5 |
